# The E1A-Associated p400 Protein Modulates Cell Fate Decisions by the Regulation of ROS Homeostasis

**DOI:** 10.1371/journal.pgen.1000983

**Published:** 2010-06-10

**Authors:** Lise Mattera, Céline Courilleau, Gaëlle Legube, Takeshi Ueda, Rikiro Fukunaga, Martine Chevillard-Briet, Yvan Canitrot, Fabrice Escaffit, Didier Trouche

**Affiliations:** 1 Laboratoire de Biologie Cellulaire et Moléculaire du Contrôle de la Prolifération (LBCMCP), CNRS and Université de Toulouse, Toulouse, France; 2 Campbell Family Institute for Breast Cancer Research, Princess Margaret Hospital, Toronto, Canada; 3 Department of Medical Chemistry, Graduate School of Medicine, Kyoto University, Kyoto, Japan; Max-Planck-Institute of Immunobiology, Germany

## Abstract

The p400 E1A-associated protein, which mediates H2A.Z incorporation at specific promoters, plays a major role in cell fate decisions: it promotes cell cycle progression and inhibits induction of apoptosis or senescence. Here, we show that p400 expression is required for the correct control of ROS metabolism. Depletion of p400 indeed increases intracellular ROS levels and causes the appearance of DNA damage, indicating that p400 maintains oxidative stress below a threshold at which DNA damages occur. Suppression of the DNA damage response using a siRNA against ATM inhibits the effects of p400 on cell cycle progression, apoptosis, or senescence, demonstrating the importance of ATM–dependent DDR pathways in cell fates control by p400. Finally, we show that these effects of p400 are dependent on direct transcriptional regulation of specific promoters and may also involve a positive feedback loop between oxidative stress and DNA breaks since we found that persistent DNA breaks are sufficient to increase ROS levels. Altogether, our results uncover an unexpected link between p400 and ROS metabolism and allow deciphering the molecular mechanisms largely responsible for cell proliferation control by p400.

## Author Summary

External or internal causes can lead to the generation of oxidative stress in mammalian cells. This oxidative stress is detrimental to cell life since it can induce protein damages or, even worse, DNA damages. Thus, cells have to control strictly oxidative stress levels. In this manuscript, we show that the p400 ATPase, a chaperone of specific histone H2A variants, is important for this control in mammals and therefore prevents DNA damage induction. Moreover, we demonstrate that the known roles of p400 in cell proliferation are dependent upon its effect on oxidative stress. Finally, we identify the mechanisms by which p400 modulates oxidative stress levels. Altogether, our study uncovers a new role of mammalian p400 and demonstrates its functional importance.

## Introduction

Cell fate decisions largely rely on the activation or the repression of specific genetic programs. Proteins, which regulate these genetic programs, are involved in the accurate control of cell fate. Among these proteins, chromatin modifying-enzymes are proposed to play a special role because they can set up epigenetic imprints in chromatin and thus mediate long term and transmissible effects on chromatin function. In mammals, one such protein is the p400 ATPase which is an ATPase of the SWI/SNF family conserved from yeast to human (it is called SWR1 in yeast and Domino in drosophila) [1]–[3]. It belongs to a multimolecular complex, which contains other enzymes such as the helicases Tip49a and Tip49b and, at least in mammals and in drosophila, the histone acetyl transferase Tip60 [1], [4]–[6]. p400 can mediate exchange of histone H2A variants, such as H2A.Z in yeast and mammals and H2Av (which is a drosophila-specific variant related to both H2A.Z and H2A.X) in drosophila [4]–[8]. Through this activity, p400 participates in various processes such as DNA double strand breaks (DSBs) repair and transcription: in drosophila, Domino exchanges phosphorylated H2Av by unphosphorylated H2Av following completion of DNA repair, leading to the suppression of DNA DSB signalling [5]. Transcriptional regulation by p400 largely relies on H2A.Z incorporation at specific promoters [9]. H2A.Z incorporation can lead both to positive or negative outcome for transcription: whereas removal of H2A.Z is often required for transcription to occur, H2A.Z can also “poise” genes for activation, preventing the propagation of neighbouring repressive heterochromatin [10]. In agreement with this dual effect of H2A.Z in transcription, p400 mediates transcriptional repression of the *p21* gene in the absence of DNA damage [11], [12] but it is also required for transcriptional activation of estrogen-responsive genes upon hormone treatment [13], both effects being mediated through H2A.Z incorporation [7].

Many results underline the role of p400 and p400-associated proteins in cell fate decisions control. First, p400 was characterized as a protein associated with the viral transforming protein E1A from adenovirus [1]. Moreover, association with p400 was found to be required for E1A to promote cell transformation as well as apoptosis [1], [14], indicating that p400 is important for E1A-mediated cell proliferation and cell transformation control. p400 prevents cell cycle arrest in human osteosarcoma-derived cells [12], inhibits apoptosis in colon carcinoma-derived cells [15] and blocks senescence induction in non transformed human fibroblasts [11] or mouse embryonic fibroblasts [16]. Also, depletion of p400 or of associated proteins (such as Tip60) results in a decrease cell proliferation rate of embryonic stem cells [17]. Altogether, these data point to a critical role of p400 in allowing cell proliferation.

The function of p400 in preventing cell cycle arrest or senescence is proposed to be mediated through the direct transcriptional regulation of p21 expression by localized H2A.Z incorporation [7]. However, we show here that p400 depletion can induce oxidative stress suggesting that it may also indirectly activate p21 expression through the activation of DNA damage pathways. By inhibiting these pathways, we show that this indirect mechanism largely account for p21 regulation by p400 as well as for downstream control of cell fate (such as senescence, cell cycle progression or apoptosis). Altogether, our results allow us to decipher the molecular mechanism which accounts for most of the effects of p400 on cell proliferation.

## Results

### p400 knockdown induces ROS accumulation

**Figure 1:**
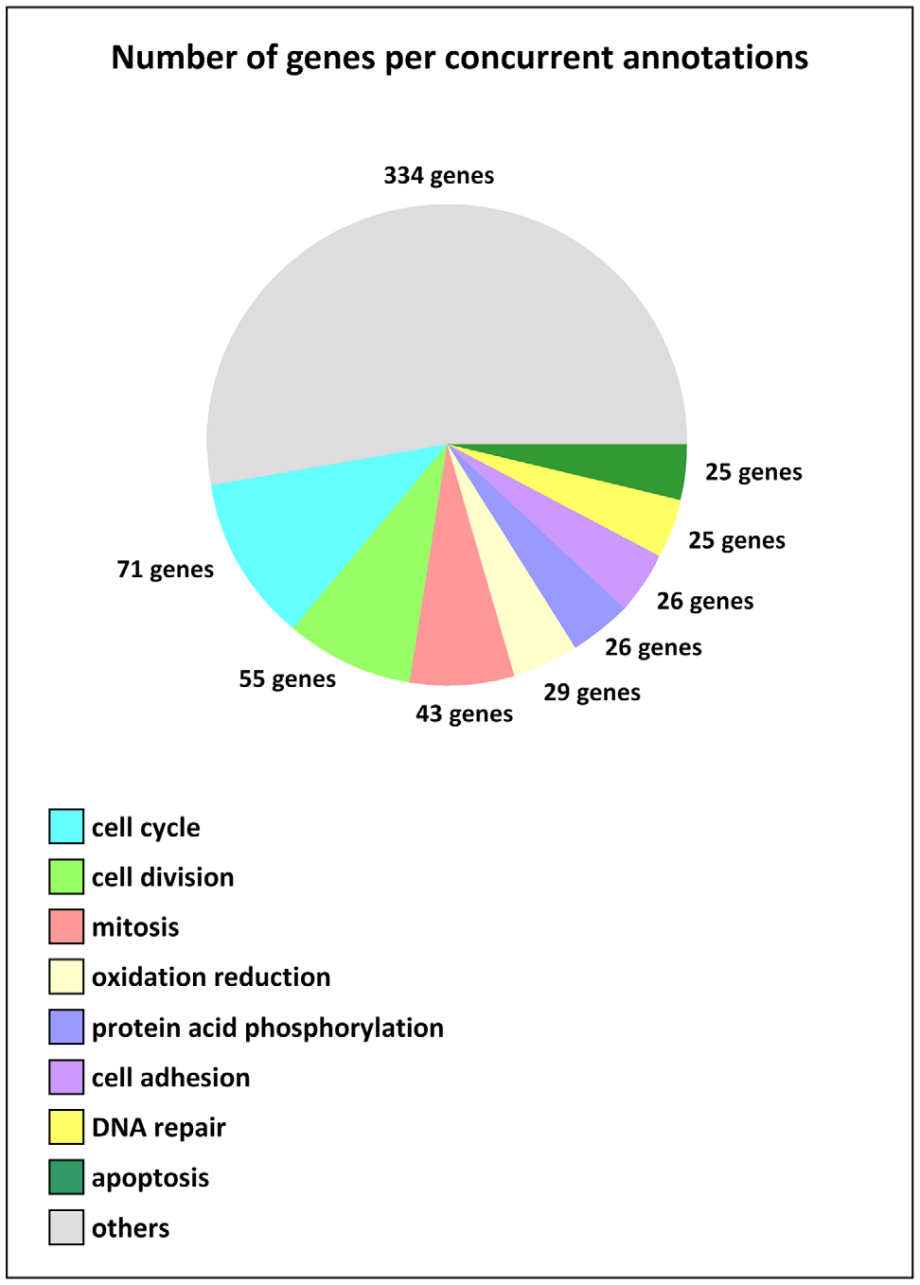
Gene singular annotation of DNA microarrays data. U2OS cells were independently transfected using two controls and two different p400-targetting siRNAs. Total RNA was then prepared 48 h later and submitted to reverse transcription, cDNA control quality and hybridization on Affymetrix DNA microarrays (GeneChip Human Gene 1.0 ST Array). Following quantification of two independent experiments, normalization and statistical analysis was done using GeneSpring GX software (Agilent Technologies). Qualified genes were those found regulated, in the same way, in at least 3 out of the four comparisons (p400-targeting siRNAs *versus* controls) and with a fold change at least equal at 1.25. Presented gene annotation discovery was done using GeneCoDis2 online software.

In order to identify genes regulated by p400, we performed a genome wide analysis of genes affected upon p400 knockdown. We transfected U2OS osteosarcoma cells in duplicates with two control siRNAs and two previously described siRNAs directed against p400 [12]. Silencing efficiency was checked by real time PCR and western blotting (Figure S1A and S1B). Moreover, the p21 mRNA was induced by p400 depletion (Figure S3B), as already described [12]. Forty-eight hours following transfection, we prepared total RNA that we analysed on Affymetrix gene microarrays containing 19,734 gene probe sets. We then compared gene expression upon transfection of each anti-p400 siRNA against each control siRNA independently. After normalization, statistical analysis and thresholding (see Materials and Methods), we considered genes as regulated by p400 when they are similarly affected in three out of the four conditions (p400-1 vs Ctrl-1, p400-2 vs Ctrl-1, p400-1 vs Ctrl-2 and p400-2 vs Ctrl-2). 878 genes were identified and, as expected, the p400 mRNA was found decreased upon p400 siRNA transfection whereas the p21 mRNA was found activated. The complete list of modified genes, sorted by the mean of fold change, is shown in Figure S2. In addition, we validated our microarrays results by analysing several genes by real time PCR (Figure S3). A Gene Ontology (GO) analysis (see Figure 1 for the eight top singular annotations) indicates that p400 mainly regulates genes linked to cell proliferation (the first three singular annotations are composed of genes involved in cell cycle, cell division and mitosis). Such regulation is expected and is likely to be indirect, since p400 knockdown affects cell cycle progression [12]. The next singular annotation represents genes involved in the oxido-reduction balance, suggesting a link between p400 and the control of oxidative stress.

**Figure 2:**
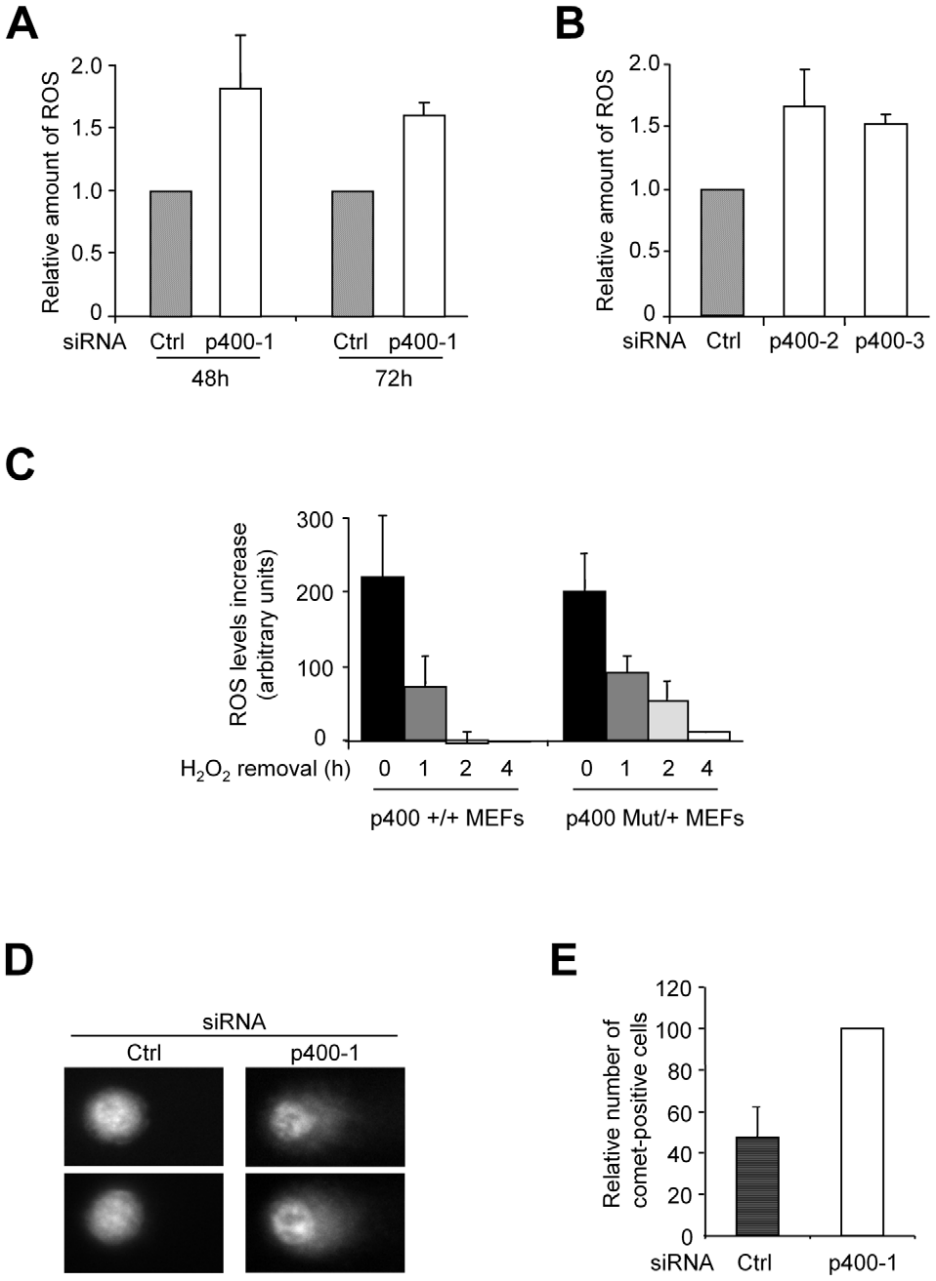
p400 depletion leads to ROS accumulation and DNA damage induction. (A) U2OS cells were transfected using the indicated siRNA. 48 and 72 h following transfection (as indicated), cells were collected and ROS levels were measured by flow cytometry. The mean and standard deviation (SD) from 3 independent experiments are shown (after standardisation relative to 1 for cells transfected with the control siRNA). (B) U2OS cells were transfected using the indicated siRNA and 48 h following transfection, cells were collected and ROS levels were measured by flow cytometry. The mean and SD from 3 independent experiments are shown (after standardisation relative to 1 for cells transfected with the control siRNA). (C) MEFs derived from heterozygous embryos in which one p400 allele was targeted (p400Mut/+)[19] or from control wild type embryos (wt) were treated or not, as indicated, with 10 mM of H_2_O_2_ for 15 min. H_2_O_2_ was washed out and cells were collected after the indicated time. ROS levels were measured by flow cytometry. The ROS levels increase was calculated by subtracting ROS levels in untreated cells (measured in parallel). The mean and SD from 2 to 3 independent experiments are shown (after standardisation relative to 1 for untreated cells). (D,E) U2OS cells were transfected using the indicated siRNA and 48 hours following transfection, cells were subjected to comet assay. Representative cells are shown in (D). The mean and SD of the proportion of comet-positive cells (tail moment >5) from three independent experiments are shown in (E) (calculated relative to 100 for cells transfected with the p400-1 siRNA).

To test this hypothesis, we directly measured the intracellular levels of ROS (Reactive Oxygen Species, a major intracellular inducer of oxidative stress) using fluorescent probes by flow cytometry upon siRNA transfection. We found that the knockdown of p400 leads to an increase of ROS levels (measured by calculating the mean fluorescence from 25,000 cells) detectable 48 hours and 72 hours following siRNA transfection (Figure 2A). This increase is similar to what is observed in cells treated with H_2_O_2_, an oxidant molecule (Figure S4A), or in cells presenting mutations in the succinate dehydrogenase enzyme, a protein directly involved in the control of the respiratory chain in mitochondria [18]. Moreover, this increase was also observed using 2 other independent p400 siRNAs (Figure 2B, see Figure S1B for RT-PCR monitoring p400 expression silencing), ruling out the possibility of any off-target effects. Thus, altogether, these results indicate that p400 is required to decrease ROS levels in U2OS cells. To confirm the importance of p400 in controlling ROS levels, we used MEFs originating from mice in which the p400 gene has been genetically targeted [19]. We prepared wild type and heterozygous MEFs. Both MEFs grow at identical rates and presented similar basal ROS levels (data not shown). We next treated these MEFs with H_2_O_2_ to analyse their response to an oxidative stress increase. We found that the increase in ROS levels following 15 min H_2_O_2_ treatment was similar in both genotypes (Figure 2C). To test their ability to cope with this ROS increase, we washed H_2_O_2_ and we analysed the decrease in ROS levels. In wild type cells, the decrease in ROS levels was very rapid since they went down to basal levels in less than two hours (Figure 2C). In MEFs in which one allele of p400 had been targeted, the decrease was slower and ROS levels was still largely above basal levels two hours following H_2_O_2_ removal (Figure 2C). Thus, the loss of one p400 allele leads to a defective management of ROS levels following a burst of oxidative stress, demonstrating that the role of p400 in controlling ROS levels is not limited to human cells neither to tumoral cells. Taken together, Figure 2A–2C data indicate that normal p400 expression is required to control ROS levels.

### p400 prevents oxidative stress-induced DNA damage and DNA damage signalling

Deregulated ROS production can lead to DNA damage. We then reasoned that p400 could maintain ROS levels below a threshold which could be detrimental to DNA integrity. To test this possibility, we investigated whether knockdown of p400 in U2OS cells leads to the generation of DNA damage. We performed a neutral comet assay, which directly reveals the amount of DNA strand breaks (single and double strand breaks) and alkali-labile sites. We found that the number of cells with detectable comet tails (thus, with detectable DNA breaks) was increased upon p400 knockdown (Figure 2D for typical cells and Figure 2E for the quantification; see Figure S5 for more detailed distribution of comet tail moments). Importantly, very similar results were obtained using another independent p400 siRNA (Figure S6). We thus conclude from this experiment that p400 expression is required for genetic integrity and that p400 prevents both increased ROS production and accumulation of DNA damage.

**Figure 3:**
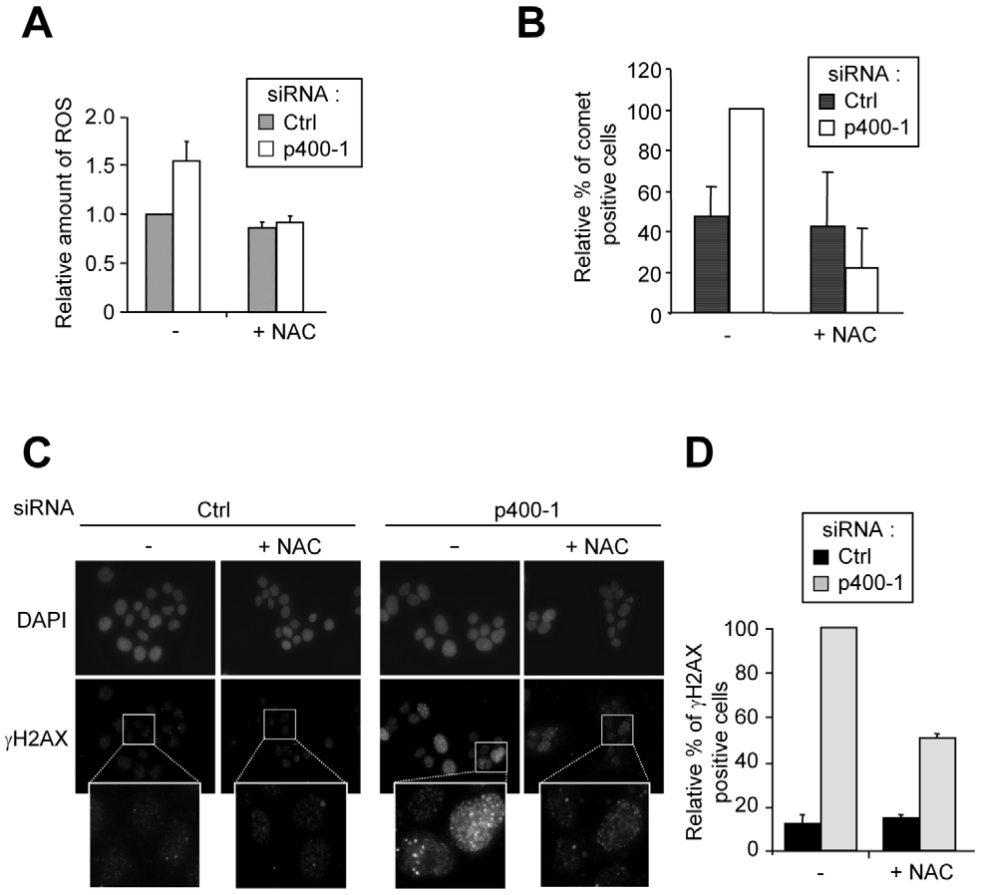
p400 prevents ROS–induced DNA damage. (A) U2OS cells were transfected using the indicated siRNA and treated with NAC (10 mM). 48 h later, cells were collected and ROS levels were measured by flow cytometry. The mean from 2 independent experiments is shown (after standardisation relative to 1 for cells transfected with the control siRNA and untreated). (B) U2OS cells were transfected using the indicated siRNA and treated with NAC (10 mM) 24 h later. 48 h following transfection, cells were subjected to a comet assay. The mean and SD of the proportion of comet-positive cells (tail moment >5) from three independent experiments are shown (calculated relative to 100 for cells transfected with the p400-1 siRNA and not treated). (C,D) Same as in (A) except that cells were fixed and subjected to DAPI staining and an immunofluorescence analysis using anti-γH2AX antibodies. Representative fields are shown in (C) and quantification using ImageJ software is shown in (D) (mean and SD from 3 independent experiments are shown (after standardisation relative to 100 for cells transfected with the p400 siRNA and untreated)).

In order to test whether DNA damage induced by the absence of p400 are due to oxidative stress, we used N-Acetyl Cysteine (NAC), a widely used anti-oxidant reagent. As expected, NAC treatment efficiently reversed the increase in ROS levels induced by p400 knockdown (Figure 3A). Importantly, NAC treatment also reversed the increase in comet tail moments upon p400 knockdown (Figure 3B, see also Figure S6 with an independent p400 siRNA). Thus, these data indicate that p400 prevents oxidative stress–induced DNA damage.

**Figure 4:**
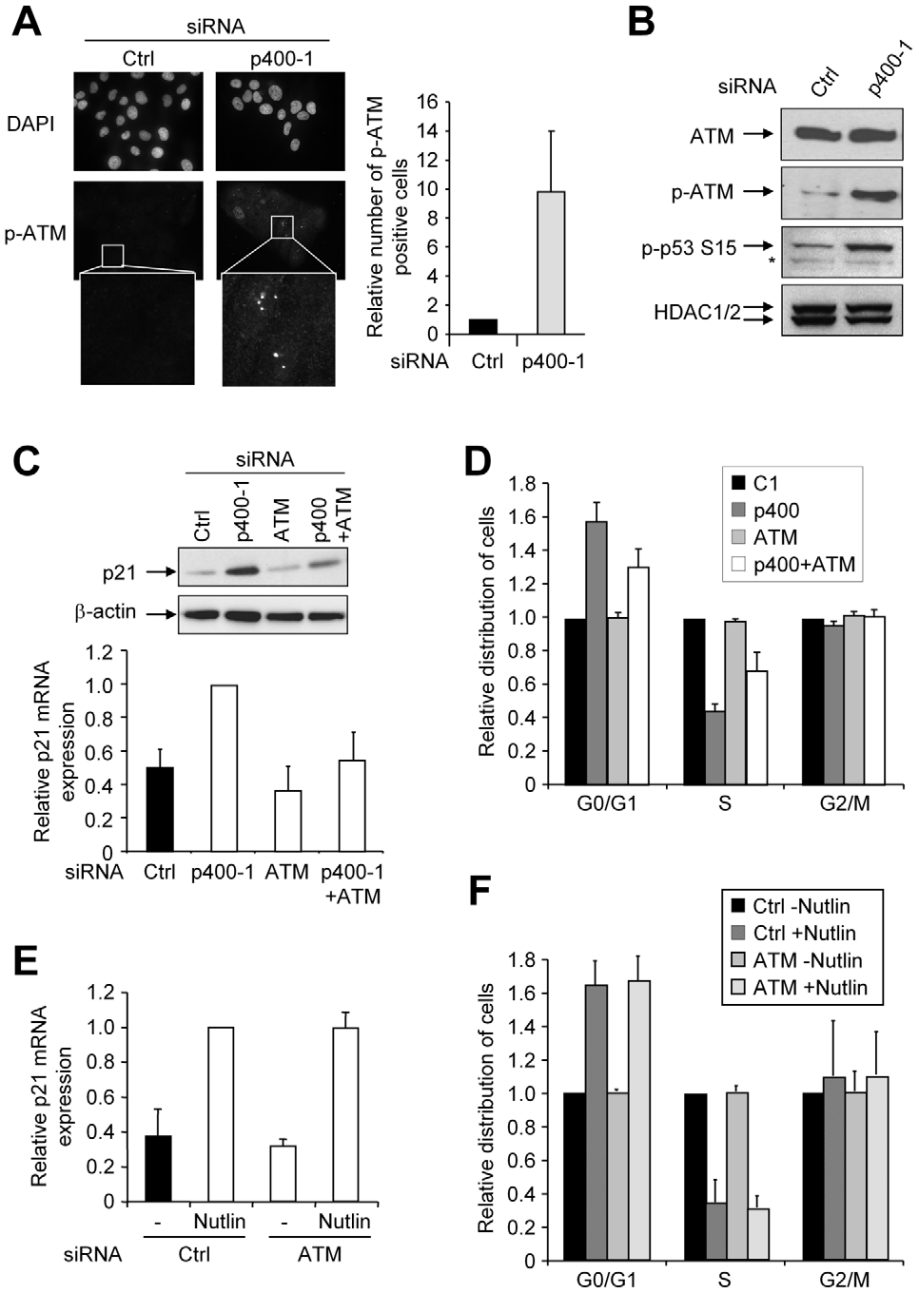
p400 depletion leads to the induction of a DNA damage response. (A) U2OS cells were transfected using the indicated siRNA. 48 h after transfection, cells were fixed and subjected to DAPI staining and an immunofluorescence analysis using anti-phosphoATM antibodies. Representative fields are shown on the left and quantification using ImageJ software on the right (the mean and SD from 3 independent experiments are shown (after standardisation relative to 1 for cells transfected with the control siRNA)). (B) Same as in (A), except that total cell extracts were prepared and analysed by western blot using anti-ATM, anti-Phospho-ATM, anti-Phospho-p53 and anti-HDAC1/2 (as a loading control) antibodies. Note that the asterisk indicates non-specific bands in the Phospho-p53 panel. (C) U2OS cells were transfected using the indicated siRNA alone or in combination as indicated. The total amount of siRNA in the transfection was kept constant using the control siRNA. 48 h later, total cell extracts were prepared and analysed by western blot using anti-p21 and anti-β-actin (as a loading control) antibodies (upper panel). Total RNA were also prepared, reverse transcribed and cDNA were then analysed for p21 and GAPDH expression by Q-PCR. The amount of p21 cDNA was divided by the amount of GAPDH cDNA and calculated relative to 1 for cells transfected using the control siRNA (lower panel). The mean and SD from 5 independent experiments are shown. (D) Same as in (C), except that cells were harvested and analysed for cell cycle distribution by flow cytometry. The proportion of G0/G1, S and G2/M cells in each condition were quantified relative to 1 for cells transfected by the control siRNA. The mean and SD from 3 independent experiments are shown. (E) U2OS were transfected using the indicated siRNA and treated with Nutlin-3. 48 h later, total RNA were prepared, reverse transcribed. cDNAs were then analysed for p21 and GAPDH expression by Q-PCR. The amount of p21 cDNA was divided by the amount of GAPDH cDNA and calculated relative to 1 for cells transfected using the control siRNA and treated with nutlin. The mean from two independent experiments is shown. (F) Same as in (E), except that cells were harvested and analysed for cell cycle distribution by flow cytometry as described in (D). The mean from two independent experiments is shown.

We next tested whether p400-induced DNA damage can lead to the activation of a DNA Damage Response (DDR) and then to a cellular response. We found that knockdown of p400 in U2OS cells is sufficient to induce a significant increase of cells harbouring γH2AX foci (Figure 3C and 3D), a widely used marker of DNA damage signalling. It also leads to an increase in autophosphorylation of the sensor kinase ATM (reflecting ATM activation), measured either by immunofluorescence or by western blot (Figure 4A and 4B). In addition, phosphorylation of downstream substrates of ATM, such as the p53 tumour suppressor, was induced (Figure 4B). Thus, p400 knockdown results in the activation of ATM-dependent DNA damage response pathways. Strikingly, NAC treatment partially reversed the appearance of γH2AX foci (Figure 3C and 3D) thereby indicating that DNA damage signalling induced by p400 siRNA is mediated through increased oxidative stress. Thus, altogether, Figure 3 and Figure 4 results indicate that p400 prevents the induction of DNA damage and of ATM-dependent DNA damage signalling by oxidative stress.

### p400 allows cell cycle progression by preventing the activation of ATM–dependent DDR pathways

We next intended to investigate the contribution of these ATM-dependent DNA damage response pathways to cell proliferation control by p400. As already reported [11], [12], we found that p400 knockdown induces the activation of the gene encoding the p21 cell cycle-dependent kinase inhibitor (Figure 4C), as well as the concomitant accumulation of cells in the G1 phase of the cell cycle (Figure 4D). Importantly, the two fold induction of p21 mRNA induction, as well as the extent of cell cycle arrest we observed here, is within the range of what has been found previously in U2OS cells by others and us [11], [12]. p400-mediated repression of the *p21* promoter was proposed to be direct and to rely on the targeted incorporation of H2A.Z on the *p21* promoter [7]. However, it may also be an indirect consequence of ATM-dependent DDR pathways repression by p400 since the *p21* promoter is a direct target of the DNA damage-activated p53 tumour suppressor. To assess the relative contribution of these two mechanisms (direct repression through H2A.Z incorporation (as demonstrated by [7]) and control of ATM-dependent DNA damage pathways (as we observed here)), we intended to inhibit these DNA damage pathways using a siRNA directed against ATM. Transfection of the ATM siRNA efficiently inhibits ATM expression and does not affect silencing of p400 by the p400 siRNA (Figure S1A). The ATM siRNA by itself does induce only a slight decrease, if any, of p21 mRNA expression (Figure 4C). However, it decreases the activation of p21 mRNA and protein expression induced by p400 depletion (Figure 4C). Moreover, it partially reversed the concomitant cell cycle arrest (Figure 4D). Importantly, activation of p21 mRNA (Figure 4E) and cell cycle arrest (Figure 4F) induced by Nutlin-3, an inhibitor of the p53/Mdm2 interaction [20], is not affected by ATM knockdown, indicating that ATM is not generally required for the p53-dependent activation of p21 mRNA expression and cell cycle arrest and then, that the effect of ATM is upstream of p53 activation. Thus, taken together, these data indicate that p400 represses p21 expression, at least in part, through the control of DNA damage pathways. Note that the residual activation of p21 mRNA and G1 accumulation induced by p400 depletion in the presence of ATM siRNA, is probably due to directs effects of p400 on the *p21* promoter [7].

### p400 controls cell fate through the modulation of DDR pathways

**Figure 5:**
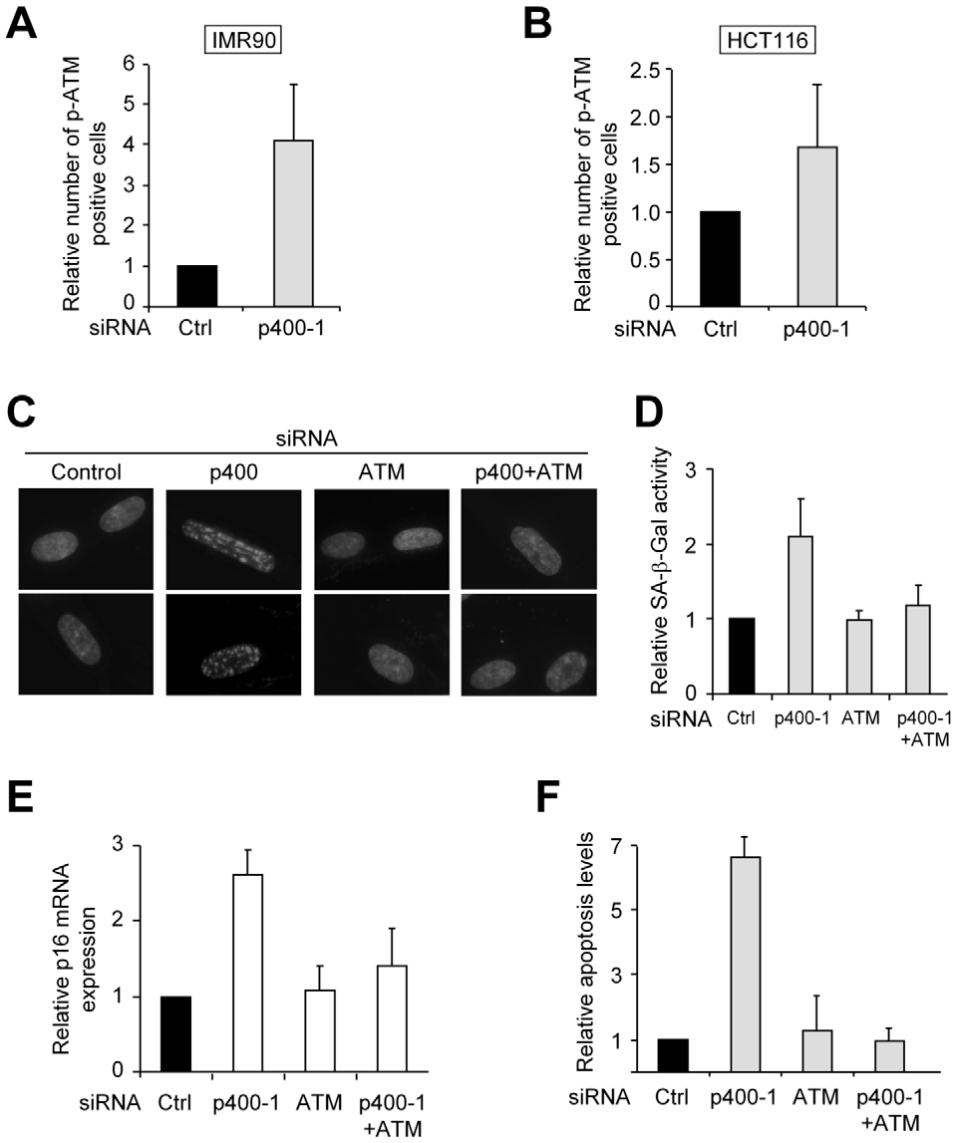
The ATM–dependent DDR is required for senescence and apoptosis induced by p400. (A,B) IMR 90 cells (A) or HCT 116 cells (B) were transfected using the indicated siRNA. 48 h following transfection, cells were fixed and analysed by immunofluorescence using an anti-phosphoATM antibody. PhosphoATM positive cells were quantified using ImageJ software and calculated relative to 1 for cells transfected with the control siRNA. The mean and SD from three independent experiments are shown. (C) IMR 90 cells were transfected with the indicated siRNA alone or in combination. The total amount of siRNA in the transfection was kept constant using the control siRNA. 7 days following transfection, cells were collected and subjected to DAPI staining to analyse SAHF formation. (D) Same as in (C), except that Senescence associated β-galactosidase activity was measured and calculated relative to 1 for cells transfected with the control siRNA alone. The mean and SD from three independent experiments are shown. (E) Same as in (C) except that mRNA were extracted and reverse transcribed. cDNAs were then analysed by Q-PCR for p16 and GAPDH mRNA expression. The amount of p16 cDNA was divided by the amount of GAPDH cDNA and calculated relative to 1 for cells transfected using the control siRNA alone. The mean and SD from three independent experiments are shown. (F) HCT116 cells were transfected with the indicated siRNA alone or in combination. The total amount of siRNA in the transfection was kept constant using the control siRNA. 48 h following transfection, apoptosis induction was measured by flow cytometry and the percentage of apoptotic cells was calculated relative to 1 for cells transfected with the control siRNA. The mean and SD from three independent experiments are shown.

Strikingly, other proposed roles of p400 in cell proliferation (repressor of apoptosis in HCT116 cells [15] and repressor of senescence in IMR90 cells [11]) could also be dependent on its ability to modulate ATM-dependent DNA damage pathways. First, we checked whether p400 also controls ATM-dependent DDR pathways in these cells by transfecting them with a p400 siRNA, which decreased p400 mRNA or protein levels as expected (Figure S7). We observed by immunofluorescence staining that p400 knockdown induces ATM phosphorylation both in IMR90 cells (Figure 5A) and HCT116 cells (Figure 5B). Thus the ability of p400 to prevent activation of DDR pathways is not restricted to U2OS cells.

We next investigated the involvement of ATM-dependent DDR pathways activation in cell fate control by p400. To this aim, we co-transfected cells with the siRNAs against p400 and ATM in HCT116 and IMR90 cells. In these two cell types, transfection of the ATM siRNA efficiently inhibits ATM expression and does not affect silencing of p400 by the p400 siRNA (Figure S7). As already demonstrated [11], transfection of p400 siRNA leads to senescence of IMR90 cells, as observed by the appearance of the so-called SAHF (Senescence-Associated Heterochromatin Foci) (Figure 5C), the induction of Senescence–Associated β-Galactosidase activity (Figure 5D) and induction of p16 mRNA expression (Figure 5E). Depletion of ATM does not have any effect by itself on senescence induction. However, depletion of ATM completely reverses the induction of senescence by the transfection of p400 siRNA (Figure 5C–5E), indicating that ATM expression is required for p400 knockdown to induce senescence. Similarly, we found that ATM expression is also required for p400 knock-down-induced apoptosis in HCT116 cells (Figure 5F). Thus taken together, these data indicate that cell fate control by p400 is largely dependent on its ability to prevent activation of ATM-dependent DNA damage pathways.

### DNA double-strand breaks induction results in ROS increase

**Figure 6:**
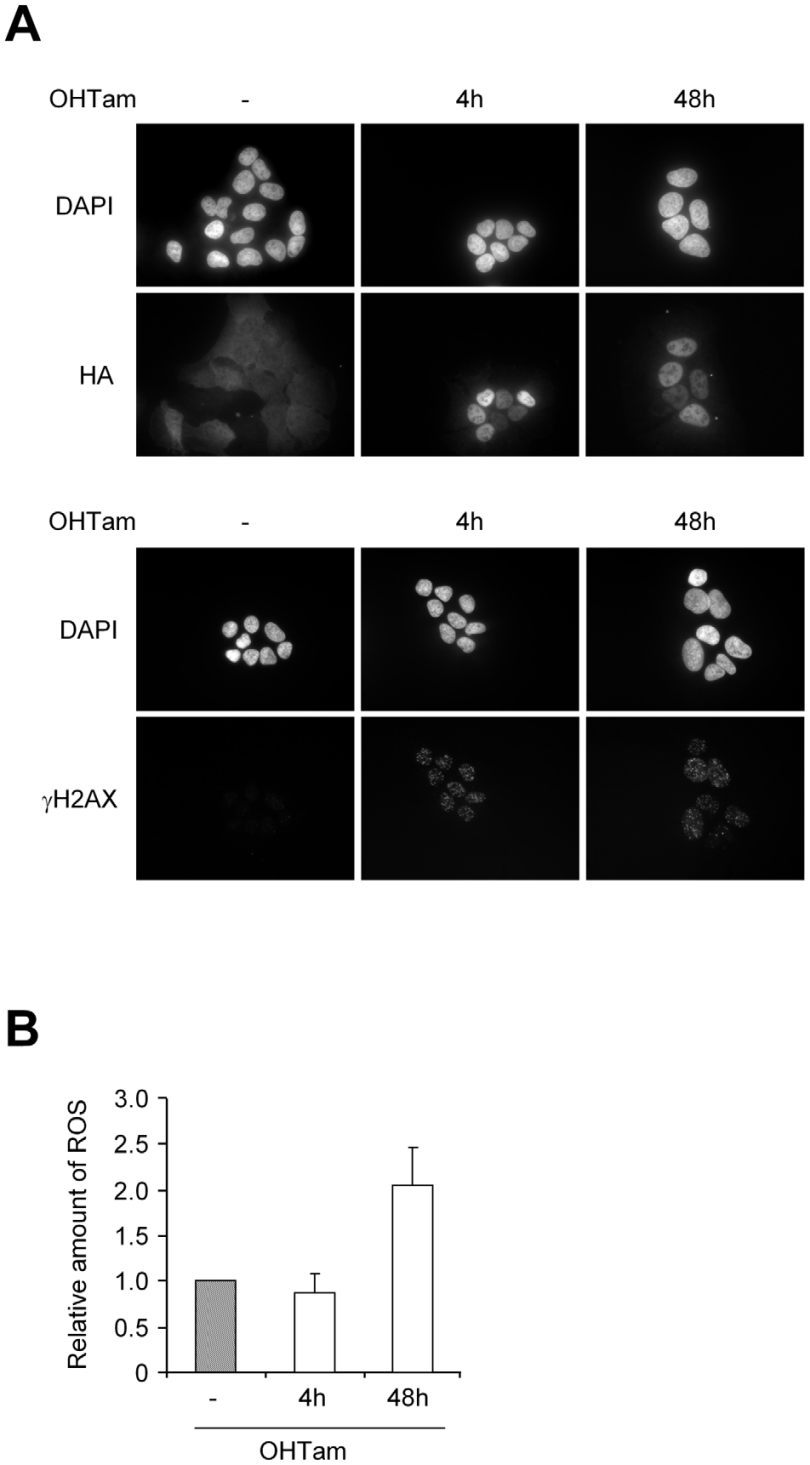
Persistent DNA breaks are sufficient to induce oxidative stress. (A) U2OS HA-AsiSI-ER cells were treated with OHTam for 4 h or 48 hours, or not, as indicated. Cells were then fixed and subjected to DAPI staining and immunofluorescence using anti-HA (top panels) and anti-γH2AX (bottom panel) antibodies. (B) Same as in (A) except that ROS levels were measured by flow cytometry. The mean and SD from 3 independent experiments are shown (after standardisation relative to 1 for cells untreated with OHTam).

We next addressed the mechanism by which p400 knockdown leads to ROS production. Strikingly more and more evidence suggests that persistent DNA damage (due for example to defective DNA repair pathways) induces an oxidative stress [21]–[24]. Since p400 depleted cells present an increase in DNA breaks (see Figure 2D and 2E), we tested whether this increase in DNA damage could participate in ROS production. To test whether DNA breaks can induce ROS production in U2OS cells, we used a cell line derived from U2OS cells in which we can induce the nuclear localisation of the AsiS1 restriction enzyme by 4-hydroxy-tamoxifen (OHTam) treatment. When localized in the nucleus, this restriction enzyme generates a large number (about 200) of pure double strand breaks (DSBs) [25] (in contrast to more widely used methods, such as ionizing radiations, which directly produce free radicals). OHTam treatment generated nuclear accumulation of the restriction enzyme and efficiently induced DNA DSBs, as indicated by the appearance of γH2AX foci (Figure 6A). DNA breaks induction is very rapid since increased γH2AX staining can be detected as early as 15 min following OHTam addition (data not shown). Moreover, very high levels of γH2AX staining are still observed 48 hours following OHTam addition demonstrating that the DNA DSBs persist up to 48 hours of treatment (most likely because restriction sites are permanently repaired and re-cleaved) (Figure 6A). We then treated, or not, these cells with OHTam for 4 or 48 hours and we measured ROS levels. Whereas no change could be detected 4 hours following OHTam, ROS levels were strongly increased after 48 hours of OHTam treatment, reaching a level similar to the one measured 48 hours after p400-targeting siRNA transfection (Figure 6B). Importantly, no increase in ROS production could be observed in parental U2OS cells treated with OHTam (data not shown), indicating that ROS production is indeed due to the generation of DSBs by the restriction enzyme. These results indicate that in U2OS, whereas the presence of DNA DSBs *per se* is not able to induce a detectable increase in ROS levels (as indicated by the results obtained 4 hours following OHTam treatment), their persistence for up to 48 hours is sufficient to induce such an increase. Thus, the presence of persistent DNA damage in cells depleted by p400 could participate in the increase in oxidative stress.

### p400 directly regulates genes important for the control of oxidative stress

**Figure 7:**
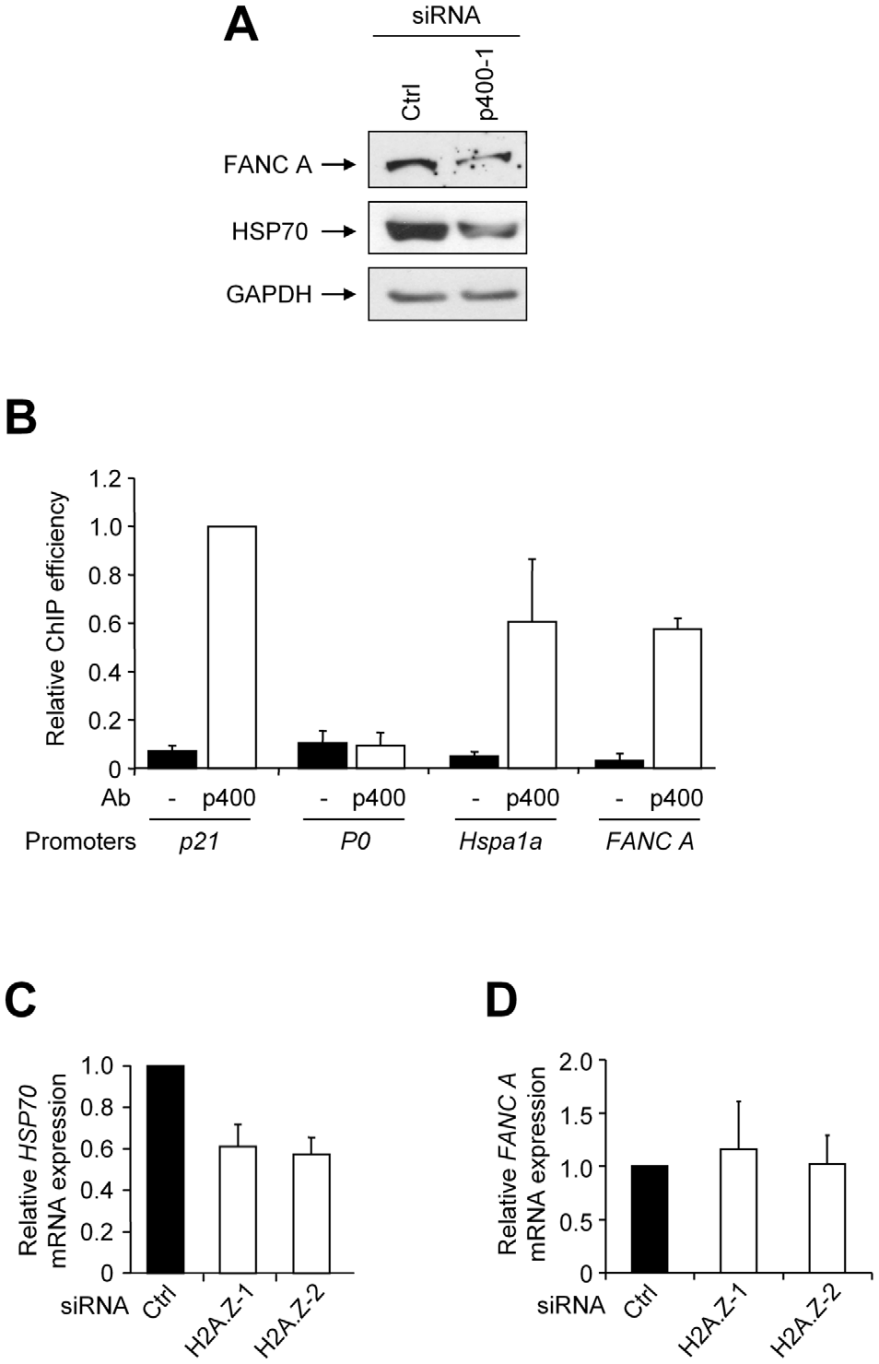
Hsp70 and FANCA are direct target genes of p400. (A) U2OS cells were transfected using the indicated siRNA. 48 h following transfection, total cell extracts were prepared and analysed by western blot using anti-FANCA, anti-Hsp70 and anti-GAPDH (as a loading control) antibodies. (B) U2OS cells were subjected to a ChIP experiment using p400 antibodies or without antibody as indicated. The amounts of *p21, Phosphoprotein P0*, *Hspa1a and FANCA* promoters in the immunoprecipitates and in the inputs were measured by Q-PCR. ChIP efficiency (% of input DNA) was calculated relative to 1 for the amount of *p21* promoter immunoprecipitated by the p400 antibody. The mean and SD from three independent experiments are plotted. (C) U2OS cells were transfected using the indicated siRNA. 48 h following transfection, cells were harvested and total RNA was prepared and reverse transcribed. cDNAs were then analysed for Hsp70 and GAPDH expression by Q-PCR. The amount of Hsp70 cDNA was divided by the amount of GAPDH cDNA and calculated relative to 1 for cells transfected using the control siRNA. The mean and SD from three independent experiments are shown. (D) Same as in (C) except that cDNA were analysed for FANCA and GAPDH expression.

However, such a mechanism does not fully explain our results since we found that DNA damage induction by p400 is partially blocked by anti oxidant treatment (indicating that DNA breaks induction is, at least partly, a consequence and not a cause of oxidative stress increase). Moreover, we found that p400 expression is important to restore normal ROS levels upon exogenous oxidative stress increase at time points (4 hours, see Figure 2C) at which DNA damages do not increase ROS levels (see Figure 6B). Thus, we investigate whether some of the effects of p400 on ROS levels control could be transcriptional. Specific inspection of the microarrays results indicates that p400 indeed regulates many genes whose products are known to be involved in the control of ROS levels, in such way that it could favour an increase in ROS levels (such as *Hsp70* [26]–[28], *FANCA* [29], [30] or *Lamin B1* [31]). To investigate the importance of transcriptional control in the effects of p400 on ROS levels, we focused on *Hsp70* and *FANCA*. Indeed, knocking-down Hsp70 expression induces oxidative stress in many cell type, and oxidative stress management is defective in cells from the Fanconi anemia group A [29], [30]. Moreover, we observed, in our microarrays analyses, that FANCA mRNA as well as two mRNAs coding for Hsp70 protein (Hspa1a and Hspa1b) (collectively referred as “Hsp70” thereafter since they encode identical proteins [32]) are decreased upon p400 knockdown (Figure S2). We first checked whether *Hsp70* and *FANCA* are *bona fide* target genes of p400. We transfected U2OS cells with two independent p400 siRNAs and analysed Hsp70 and FANCA mRNA expression by RT-QPCR. We confirmed that Hsp70 and FANCA mRNA levels are decreased in cells transfected by p400 siRNA, indicating that p400 positively regulates their expression (Figure S3C). Moreover, we also found that p400 knockdown decreases Hsp70 and FANCA protein expression (Figure 7A). We next tested whether these two genes can be direct transcriptional targets of p400: indeed, p400 is known to favour transcription through binding to specific promoters, such as some controlling genes involved in maintenance of embryonic stem cells [17] or estrogen-responsive genes [13]. Thus, to test whether p400 can directly regulate the promoter of *Hspa1a* (a gene coding for Hsp70 protein) and of *FANCA*, we performed Chromatin ImmunoPrecipitation (ChIP) experiments with p400 antibodies using chromatin from U2OS cells. As expected, we could detect p400 binding to the *p21* promoter (a known direct target of p400 [11]) (Figure 7B). This binding is specific, since an unrelated sequence derived from the ribosomal phosphoprotein *P0* promoter was only marginally enriched in the p400 immunoprecipitates (IP). Strikingly, we found that sequences derived from the *Hspa1a* gene promoter and from the *FANCA* promoter are also enriched in the p400 IP (with an efficiency comparable to the *p21* promoter) (Figure 7B), indicating that p400 physically binds to these two promoters. Taken together, these results indicate that the *Hspa1a* and *FANCA* promoters are directly regulated by p400.

Direct regulation of promoter activity by p400 often involves incorporation of the H2A.Z variant. To test the involvement of H2A.Z incorporation in p400-mediated regulation of these promoters, we depleted H2A.Z expression using a specific siRNA (see Figure S1C for RT-PCR and western blot showing the efficiency of H2A.Z depletion). We found that this depletion leads to a decrease in Hsp70 mRNA expression (Figure 7C) but not of FANCA mRNA expression (Figure 7D), suggesting that p400 regulates these two promoters by independent mechanisms.

### p400-regulation of Hsp70 and FANCA is important for ROS control

**Figure 8:**
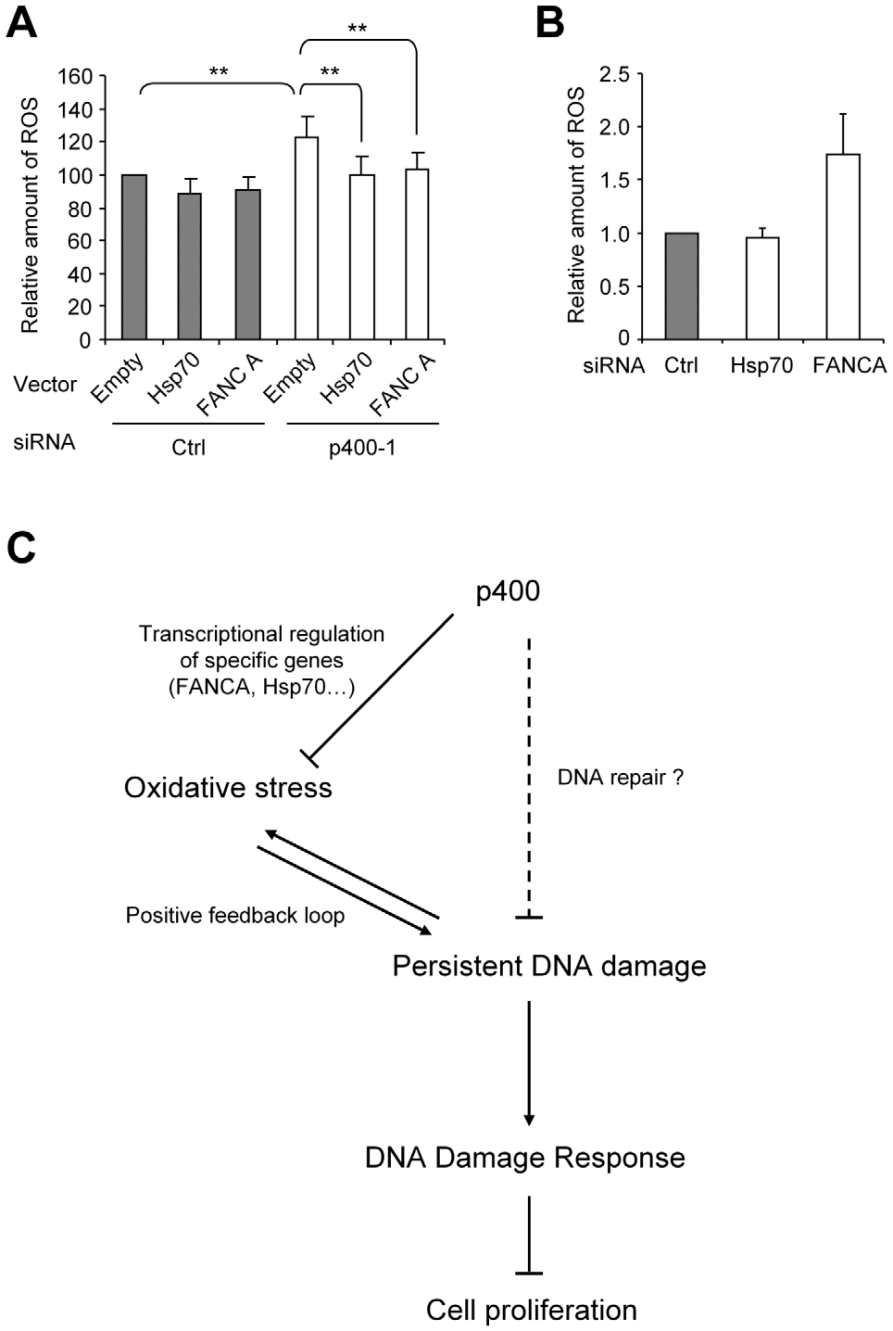
Transcriptional regulation of Hsp70 is involved in ROS controls by p400. (A) U2OS cells were transfected using the indicated siRNA and Hsp70 or FANCA expression vector (1.5 µg). Total amount of DNA in the transfection was kept constant using the corresponding empty vector. 48 h later, cells were collected and ROS levels were measured by flow cytometry and calculated relative to 1 for cells transfected by the control siRNA and empty vector. The mean and SD from three independent experiments are shown. The two stars (**) indicate a p value below 10^−5^ (Student t-test). B) U2OS cells were transfected using the indicated siRNA and 48 h following transfection, cells were collected and ROS levels were measured by flow cytometry. The mean and SD from 3 independent experiments are shown (after standardisation relative to 1 for cells transfected with the control siRNA). (C) Our model of cell proliferation control by p400: p400 prevents oxidative stress through transcriptional regulation of specific promoters. Oxidative stress in turn induces persistent DNA damage. These persistent DNA damages favour an additional oxidative stress increase (“positive feedback loop”) and induce the activation of the DNA damage response pathways, resulting in a potent inhibition of cell proliferation (through senescence, cell cycle arrest or apoptosis induction depending on the cell type). The dash line represents a putative role of p400 in DNA repair (demonstrated in other species), which may contribute to the persistence of DNA damage.

We next tested whether transcriptional regulation can be important for p400 to control intracellular ROS levels. To this aim, we transfected together with the p400 siRNA, an expression vector for Hsp70 and FANCA to prevent the decrease of their mRNA. Such an experiment is feasible since transfection efficiency for plasmids routinely reached from 50 to 80% in U2OS cells (data not shown). In addition, RT-PCR measurement of mRNA levels indicated that, in cells transfected by the expression vector, the p400 siRNA efficiency was unchanged (data not shown). We next measured ROS levels 48 hours following transfection. We found that ectopic expression of Hsp70 or FANCA have, by themselves, no effect on ROS levels in U2OS cells (Figure 8A). However, they significantly inhibited the increase in ROS levels induced by p400 knockdown (Student t-test p<10^−5^ on three independent experiments). This result indicates that, if the down regulation of Hsp70 or FANCA is prevented, p400 siRNA transfection induces ROS production less efficiently. Thus, regulation of Hsp70 and FANCA expression is required for p400 to control ROS production. Finally, we transfected U2OS cells with siRNAs directed against Hsp70 and FANCA to test whether we can recapitulate the effects of the p400 siRNA on ROS production. Both siRNAs inhibited their targets as shown by reverse transcription followed by Q-PCR or western blotting experiments (Figure S1D and S1E). We next measured their effects on ROS production and we found that, whereas inhibition of Hsp70 has no effect by itself, transfection of FANCA siRNA induces ROS accumulation (Figure 8B). Thus, this result indicates that FANCA is certainly a critical target by which p400 controls oxidative stress. In agreement with this hypothesis, H2A.Z inhibition, which does not affect FANCA expression (Figure 7D), does not induce any increase in ROS levels (Figure S8). Defects in Hsp70 expression, although probably not causal in oxidative stress induction, likely participates in the defective response to oxidative stress in p400-depleted cells, since activation of Hsp70 mRNA expression upon acute oxidative stress in MEFs is abrogated upon loss of one p400 allele (Figure S9). Taken together, these results indicate that the p400-mediated control of ROS levels and cell proliferation is brought about, at least in part, through the transcriptional regulation of specific promoters, including the *FANCA* and *Hsp70* promoters.

## Discussion

### p400 controls anti-oxidant pathways

In this manuscript, we first demonstrate that p400 plays a major role in the control of ROS metabolism since a decrease of p400 level is sufficient to induce a ROS imbalance in U2OS cells. Consequently, p400 expression is required to maintain ROS levels below a threshold at which DNA damage are induced and the DNA damage response (DDR) activated, and is important for ROS homeostasis upon a burst of oxidative stress. We further provide important information on the mechanism by which p400 exerts its anti-oxidant functions: First, we demonstrate that part of the mechanism is transcriptional: indeed, some genes known to be involved in the control of ROS levels are positively regulated by p400 such as the genes coding for FANCA, Hsp70 and Lamin B1. Moreover, the regulation of some of these proteins (FANCA and Hsp70) is required for p400 to control ROS levels. It is likely that the initial increase in ROS production upon p400 knock-down is related to transcriptional defects in the expression of genes involved in ROS metabolism such as *FANCA*. Indeed, p400 depletion leads to a decrease of FANCA levels to an extend at which FANCA knockdown induces ROS (Figure 8B). It is thus likely that the transcriptional defects, in the absence of p400, lead to a deficiency in ROS removal and thus, due to the continuous ROS production in living cells, in an increase in ROS levels and oxidative stress. This increase will then lead to the induction of DNA damage, since we found that DNA damages induction is largely reversed by anti-oxidant treatment (Figure 3B).

Interestingly, our results suggest that the continuous presence of DSBs per se is sufficient to induce ROS levels. The mechanism by which persistent DSBs induces an oxidative stress is not known but may be related to an elevated cellular metabolism to achieve DNA repair. In agreement with this hypothesis, the existence of a link between defects in DNA damage repair pathways and increased ROS production is now becoming more and more clear [21]–[24]. Whatever the mechanism, the presence of persistent DSBs in the absence of p400 may contribute to the observed increase in ROS levels. These DSBs can be continuously created or may persist because unrepaired. Strikingly, some p400 homologues in other species (SWR1 in yeast and domino in drosophila) are known to participate in DNA DSB repair. Moreover, we identify here FANCA, a protein important for DNA repair, as a direct target gene of p400. Finally, the GO analysis of our microarrays data of p400 target genes has identified DNA repair as a major singular annotation (See Figure 1). Therefore, it is tempting to speculate that when p400 levels are low, unrepaired DSBs accumulate and the persistence of these DNA breaks leads to ROS increase.

Such a mechanism cannot fully explain by itself our results since, as noted above, antioxidant treatment largely reversed DNA breaks (which would not be the case if DNA breaks would be responsible for increased ROS production) and since p400Mut/+ MEFs exhibit a defective ROS response compared to wild type MEFs at time points at which DNA breaks do not induce ROS accumulation. Taken together, our results indicate that the transcriptional defects in p400-depleted cells decrease ROS metabolism leading to an initial ROS increase. This initial ROS increase leads to the appearance of persistent DNA breaks, which in turn favour continuous ROS production, which are incorrectly managed in the absence of p400, resulting in the apparition of a positive feedback loop inducing oxidative stress and the downstream cellular response (see our model in Figure 8C).

### p400-mediated cell fate decisions largely rely on ROS induction

p400 has been shown to play a critical role in cell fate decision since it strongly favours cell proliferation by preventing senescence induction [11], cell cycle arrest [12] and apoptosis induction [15]. Our data here indicate that inhibition of these anti-proliferative states by p400 largely relies on the same molecular mechanism, which is the control of intracellular ROS levels: upon p400 depletion, ROS levels are increased leading to endogenous oxidative stress. This stress is high enough to induce DNA damage and activation of the ATM-dependent DNA Damage Response. Depending on the cell type, activation of this DDR following p400 knockdown will lead to induction of senescence (normal cells such as IMR90 cells), cell cycle arrest or apoptosis (tumoral cells). Importantly, although cell fate control by p400 was originally shown to be largely dependent on the p53 tumour suppressor [11], [12], it is now clear that the effects of p400 on senescence or apoptosis can also be p53-independent [15], [16]: these latter effects could be mediated by p53-independent DDR pathways. Also, our findings could explain why p400 and its associated protein Tip60 have been described as antagonists [12], [15]. Indeed, Tip60 is largely required for DNA damage Response [33], at least in part through ATM acetylation [34], [35]: inactivation of Tip60 would abolish any effects due to DDR activation following p400 depletion, as we described for p21 activation and accumulation of cells in G1 [12] or for apoptosis induction [15].

Of note, Gevry et al. proposed that p400 represses *p21* promoter through the targeted incorporation of H2A.Z variant and that this repression is lost upon DNA damage induction [7]. Although we were able to confirm that p400 binds to the *p21* promoter (Figure 7B) and that H2A.Z is enriched on the *p21* promoter (data not shown), we show here that p400 can repress p21 transcription indirectly: indeed, in U2OS cells, p400 knockdown induces activation of the ATM-dependent DDR pathway and phosphorylation of p53. Moreover, induction of ROS to levels comparable to those observed upon p400 depletion is sufficient to induce activation of p21 mRNA expression (Figure S4B). To distinguish between direct (through regulated H2A.Z incorporation) or indirect (through the control of the DDR pathways) repression of *p21* promoter by p400, we inhibited the DDR pathway and we found that we can partially relieve the effects of p400 knockdown. Thus, the integrity of the DDR pathway is required for full p21 activation following p400 knockdown. In contrast, the DDR pathway is not required for full p21 activation by p53 in the absence of DNA damage (by the Mdm2 inhibitor Nutlin-3, see Figure 4E). Taken together, these data demonstrate that regulation of the *p21* promoter by p400 involves, at least in part, control of the DDR pathways upstream of p53 transcriptional activity. Interestingly, such a mechanism is consistent with our previous finding that p400 depletion does not further activate p21 expression upon full induction of DDR pathways through genotoxic treatments [12]. Note however that, because ATM inhibition does not totally relieve p21 activation by p400 knockdown, we cannot rule out the possibility that H2A.Z incorporation participates in the repression of p21 expression by p400 in U2OS cells.

### p400 and the stress response

Here we identify *Hspa1a* (a gene encoding Hsp70 protein) and *FANCA* as direct target genes of p400. *Hspa1a* and *FANCA* can thus be added to known target genes of p400 which include some E2F and c-myc target genes [36], [37], the p21 cell cycle inhibitor [11], [12], estradiol-receptor target genes [13] and genes required for pluripotency maintenance in ES cells [17]. Hsp70 plays a major role in adaptation to various types of stresses [32]. p400, through the regulation of Hsp70 expression, may also be generally involved in stress response. Indeed, cells deficient for both Hsp70 proteins are more susceptible to UV, osmotic stress, ischemia, and heat [32]. It would be of particular interest to investigate whether p400 itself is a target of stress-induced pathways. Strikingly, cancer cells are subjected to various types of stress and increased Hsp70 levels is a common feature of cancer cells [38]. This increase is believed to help cancer cells to cope with the various stresses they encounter, including therapy-induced stress. Therefore, Hsp70 is an increasingly popular potential therapeutic target. The results we present here suggest that deregulation of p400 function may also favour cancer progression and resistance to anticancer treatments through the control of stress-response pathways. In agreement with such a hypothesis, we recently showed that the siRNA-mediated decrease of p400 levels favours the response to 5-fluorouracil of colon cancer cells [15]. Our data highlight the importance of studying p400 expression in human cancer and confirms that p400 could be a promising therapeutic target.

## Materials and Methods

### Antibodies, plasmids, siRNAs, and Q-PCR primers

The p400 antibody was purchased from Abcam (Paris, France), the anti-HDACs (which recognizes HDAC1, 2 and 3) from Transduction Laboratories (Lexington, KY), the anti-HA from Covance (Madison, WI), the anti-γH2AX from Upstate Biotechnologies (Millipore, Inc. Billerica, MA), the anti-GAPDH antibody from Chemicon International, Inc (Temecula, CA), the anti-ATM and the anti-phospho-ser15 p53 from Calbiochem (EMD Chemicals, Inc. Darmstadt, Germany), the anti-phospho ATM from Cell Signalling technology, Inc (Boston, MA), the anti-H2A.Z and anti-FANCA from Abcam Inc (Cambridge, MA), the anti-Hsp70 from StressGen (Ann Harbor, MI) and the anti-HA (Y-11) and anti-β-actin (C-2) from Santa Cruz Biotechnology Inc (Santa Cruz, CA). All secondary antibodies were purchased from Amersham (Piscataway, NJ).

Hsp70 expressing plasmids and the corresponding empty vector were kind gifts from Dr Claire Vourc'h. FANCA expressing plasmid was a kind gift of Dr Filippo Rosselli (IGR, Villejuif, France).

All siRNAs were purchased from Eurogentec. The control siRNA does not recognize any human mRNA. The sequences of the top strands of the various siRNAs were as follows:

Ctrl: CAUGUCAUGUGUCACAUCU-dTdT

p400-1: UGAAGAAGGUUCCCAAGAA-dTdT

p400-2: CAUCCACAUAUACAGGCUU-dTdT

p400-3: CGACACAUUGGAUACAGAA-dTdT

Hsp70: GCGAGAGGGUGUCAGCCAA-dTdT

ATM: GCCUCCAGGCAGAAAAAGA-dTdT

H2A.Z-1: GUAGUGGGUUUUGAUUGAG-dTdT

H2A.Z-2: AAAGGACAACAGAAGACUG-dTdT

FANCA: AAGCTGTCTTCCCTGTTAGAGTT-dTdT

The efficiency of siRNAs silencing was checked in each experiments by reverse transcription followed by real time PCR as described [12].

The following primer pairs were used to amplify cDNAs following reverse transcription experiments (from 5′ to 3′):

p400: CTGCTGCGAAGAAGCTCGTT and CAATTCTTTCCCTCTCCTGC

ATM: ACCACACAGGAGAATATGGA and CTCTGCAGTAATGTATTACACA

p21: GTCAGAACCGGCTGGGGATG and TGAGCGAGGCACAAGGGTAC

p16: CTGCCCAACGCACCGAATAG and ACCACCAGCGTGTCCAGGAA

Hsp70: ACCAAGCAGACGCAGATCTTC and TCGGCCAAGGTGTTGGCGTCC

H2AZ: CCTTTTCTCTGCCTTGCTTG and CGGTGAGGTACTCCAGGATG

FANCA: CCAGCGTGATGTTATATCGG and CAAGGAATCCCTCGTCCTAC

GAPDH: GAAGGTGAAGGTCGGAGTCA and GAAGATGGTGATGGGATTTC

The following primer pairs were used to amplify promoters following ChIP experiments (from 5′ to 3′):

*P21*: GTGGCTCTGATTGGCTTTCTG and CTGAAAACAGGCAGCCCAAGG

*Hspa1a*: CCGACCCTTCCTGTCAATTA and TTCCTTGGACCAATCAGAGG

*FANCA*: GTCGTGGCCATGTTGGTC and CTTCAGGACCAACCCCAGT

*P0*: GGCGACCTGGAAGTCCAACT and CCATCAGCACCACAGCCTTC

### Cell culture and transfections

Culture products were purchased from Invitrogen (Carlsbad, CA). The colorectal carcinoma cell line HCT116, the human normal lung fibroblasts IMR90 and the osteosarcoma cell line U2OS were purchased from the ATCC collection. HCT116 and U2OS cells were cultured in Dulbecco's modified Eagle's medium (DMEM) and IMR90 in Modified Eagle's medium (MEM) supplemented with antibiotics, 10% FCS and non-essential amino acids (for IMR90). The construction of U2OS-HA-AsiSI-ER cells is described in [25].

MEF cells were prepared from p400Mut/+ mice [19] using E11.5 embryos. Dissected tissues were subjected to trypsinization and dissociation using syringe before plating cells in Petri dishes containing DMEM medium supplemented with 10% FCS, Penicillin/Streptomycin cocktail and 10 µM β-mercaptoethanol. MEF cells were then genotyped using RedExtract-N-Amp Tissue PCR kit (Sigma, Saint Quentin Fallavier, France) and used in experiment or maintained in culture for a maximum of five passages.

H_2_O_2_, N-Acetyl-Cysteine (NAC) and Nutlin-3 were purchased from Sigma and were added on cells at 10 mM, 10 mM and 20 µM, respectively. When needed, U2OS-HA-AsiSI-ER cells were treated with 300 nM 4-OHTam for 4 h or 24 h. For transfection, cells were electroporated with siRNAs or plasmids using an electroporation device (Amaxa AG, Köln, Germany), according to manufacturer's specifications.

### Extract preparation and western blot analysis

Nuclear extracts or total cell lysates were prepared as previously described [15]. 10 to 50 µg of proteins per lane were separated by NuPAGE Novex 3-8% Tris-acetate gel (Invitrogen). Proteins in the gel were transferred on a PVDF membrane. Primary antibodies as well as peroxidase-conjugated secondary antibodies were used according to standard western blot procedure and peroxidase was detected by using the Lumi-Light^PLUS^ Western Blotting Substrate (Roche Diagnostics, Meylan, France).

### RNA extraction and reverse transcription

Total RNA was extracted using an RNeasy mini kit (QIAGEN). 2 µg of each purified RNA preparation was reverse-transcribed and cDNAs were analysed by Q-PCR using specific primers (see above).

### DNA microarray analysis

Analysis on Affymetrix DNA microarrays (GeneChip Human Gene 1.0 ST Array) was carried out using U2OS cells previously transfected with siRNA targeting p400 (p400-1, p400-2; previously described in [12]), or with control siRNA (C1, C2). p400-silencing efficiency was checked by real time PCR and western blotting (Figure S1A and S1B). 48 h after siRNA transfection, 100 ng of total RNA for each condition was subjected to cleanup, reverse transcription, amplification and labelling according to the manufacturer's instructions (GeneChip Whole Transcript Sense Target Labelling Assay; Affymetrix).

Following quantification, raw data normalization and statistical analysis was done using GeneSpring GX 10.0 analysis software (Agilent technologies Inc, Santa Clara, CA). Briefly, after normalization using the RMA algorithm, a T-test statistical analysis was carried out to select genes whose expression levels significantly change in knockdown p400 condition compared to controls (p-value <0.05). The Fold Change (FC) for each gene was calculated for each siRNA p400 relative to each siRNA control. Qualified genes are those found regulated with stringent criteria, i.e. when their expression was modified more than 1.25 fold, in the same way, in at least three out of four comparisons (p400-1 vs C1, p400-2 vs C1, p400-1 vs C2 and p400-2 vs C2). Validation was done by RT-QPCR using FANCA, Hsp70, TP53INP1 and p21 primers (Figure S3).

Gene co-occurrence analysis was done using GeneCoDis2 online software (http://genecodis.dacya.ucm.es/analysis/) [39], [40].

### Real-time PCR analysis

Q-PCR analysis was performed on a CFX96 Real-time system device (Biorad) using the platinium SYBR Green qPCR SuperMix (Invitrogen), according to the manufacturer's instructions, and specific primers (see above). All experiments included a standard curve and all samples were analyzed in triplicates.

### Senescence Associated β-Gal assay (SA β-Gal assay)

Cells were submitted to the SA- β-Gal assay according to the manufacturer's instructions (96-well Cellular Senescence Assay Kit, Cell Biolabs). Fluorescence was read with a fluorescence plate reader (Fluoroskan Ascent, Thermolabsystems, Courtaboeuf, France) at 365 nm (Excitation)/502 nm (Emission).

### Cell cycle and apoptosis analysis using flow cytometry

For apoptosis analysis, cells were harvested and treated using an AnnexinV-FITC/7-AAD kit (Beckman Coulter, Marseille, France) according to the manufacturer's instructions. Cells were then analyzed by flow cytometry using FACScalibur apparatus (Becton Dickinson).

For cell cycle analysis, cells were harvested, fixed with ethanol and treated 30 min with Propidium iodide. Cells were then analyzed by flow cytometry using FACScalibur and cell cycle distribution was calculated using the ModFit LT V3.0 software (Verity Software House, Inc. Topsham, ME).

### DAPI staining, immunofluorescence, and quantification

Immunofluorescence of cells on coverslips, observations and acquisition of native images were performed as previously described [41]. Quantification of fluorescence levels was done on approximately 100 cells/slide using home-developed macros in ImageJ software (NIH, Bethesda, MA) to normalize background, thresholds and signals.

### Comet assay

The presence of DNA damages was assayed by alkaline comet assay. U2OS cells were transfected with siRNA. After 48 h, cells were harvested, mixed with low-melting-point agarose and layered onto agarose-coated glass slides. Slides were maintained in the dark at 4°C until electrophoresis. Slides were submerged in lysis buffer for 1 h and incubated for 30 min in alkaline electrophoresis buffer. After electrophoresis, slides were neutralized and stained with ethidium bromide. Average Comet Tail Moment was scored for 100 cells/slide by using the CometScore-v 1.5 software.

### Measurement of intracellular ROS levels

Generation of ROS was studied by flow cytometry using carboxy difluorodihydroFDA probe (Invitrogen). Cells seeded in 6-well plates were washed with PBS and incubated with FDA probe (5 µg/ml) in HBSS solution for 15 min at 37°C. Plates were then placed on ice and trypsinized. Cells were resuspended in PBS and immediately analysed by flow cytometry using FACScalibur apparatus. The mean fluorescence intensity of 25,000 cells was analyzed in each sample and corrected for autofluorescence from unlabeled cells.

### Chromatin Immunoprecipitation experiments

Experiments were performed as previously described in [12]. Briefly, a crosslink was done using formaldehyde 1% followed by lysis of the cells, sonication of the DNA and immunoprecipitation of protein/DNA complexes with specific antibodies or without antibody as negative control. Crosslink was then reversed by adding NaCl and DNA was purified with the GFX PCR kit (Amersham) and analyzed by Q-PCR using specific primers (see above).

## Supporting Information

Figure S1 Typical experiments of siRNA-mediated silencing of p400 and/or ATM (A), p400 (B), H2A.Z (C), Hsp70 (D), and FANCA (E) in U2OS cells. U2OS cells were transfected as described throughout the manuscript. 48 hours later, total cell extracts or total RNA were prepared and analysed by western blot using specific antibodies or by QPCR after reverse transcription, respectively. The amounts of specific cDNA were divided by the amount of GAPDH cDNA and calculated relative to 1 for cells transfected by the control siRNA. A typical result is shown for each experiment and error bars stand for the variation between the three Q-PCR replicates.
(2.19 MB PPT)

Figure S2 Complete list of modified genes upon p400 knock-down, sorted by the mean of fold change.
(0.17 MB XLS)

Figure S3 Validation, by RT-QPCR, of DNA microarray data obtained after siRNA-mediated silencing of p400 in U2OS cells. U2OS cells were transfected as described throughout the manuscript. 48 hours later, total RNA were prepared and analysed by QPCR after reverse transcription. The amounts of specific cDNA were divided by the amount of GAPDH cDNA and calculated relative to 1 for cells transfected by the control siRNA. p400 knockdown efficiency (A) and results for two increased (B) and two decreased (C) genes are shown. Error bars stand for the variation between the three independent replicates.
(0.13 MB PPT)

Figure S4 Induction of ROS and p21 mRNA in U2OS by H2O2 treatment. (A) U2OS cells were treated for 15 minutes using 10 mM H2O2 in PBS. After washing with PBS, cells were incubated with the FDA probe for 15 minutes, harvested and analyzed by flow cytometry. Error bars stand for the variation between the three independent replicates. (B) U2OS cells were treated for 15 minutes using 5 mM H2O2 in PBS. After washing with PBS, cells were incubated in serum-complemented DMEM medium for 6 h at 37°C, harvested and total mRNA were extracted. After reverse-transcription, QPCR using p21 primers was performed. Error bars stand for the variation between the three independent replicates.
(0.07 MB PPT)

Figure S5 Representative experiment of Comet tails analysis. U2OS cells were transfected with siRNA. After 48 h, the presence of DNA damages was assayed by alkaline comet assay. Tail Moment was scored for 100 cells/slide. Cells with tail moments greater than 5 were considered as DNA damage positive cells.
(0.07 MB PPT)

Figure S6 Effects of the p400-2 siRNA on DNA damage U2OS cells were transfected by the indicated siRNA as described throughout the manuscript. NAC was added, or not, 24 hours later. 48 hours following transfection, cells were harvested and subjected to a comet assay. The proportions of comet-positive cells were calculated relative to 100 for cells transfected with the p400-2 siRNA in the absence of NAC.
(0.04 MB PPT)

Figure S7 Characterization of silencing efficiency of p400 and ATM siRNAs in IMR90 and HCT116 cells IMR90 and HCT116 cells were transfected as described in the manuscript. siRNA-mediated silencing was checked by western blotting and reverse transcription followed by Q-PCR as described in Figure S1. A typical result is shown and error bars stand for the variation between the three Q-PCR replicates. Note that we were unable to detect full length p400 in IMR90 cells because of a co-migrating non-specific band.
(2.37 MB PPT)

Figure S8 Induction of ROS following H2A.Z knock-down. U2OS cells were transfected using two siRNAs directed against H2A.Z and ROS levels were measured 48 h later by flow cytometry. Resultswere calculated relative to 1 for cells transfected with control siRNA. The mean and SD from 3 independent experiments are shown.
(0.07 MB PPT)

Figure S9 Hsp70 mRNA expression in MEFs following H2O2 treatment. MEFs derived from heterozygous embryos in which one p400 allele was targeted (p400Mut/+)[19] or from control wild type embryos (p400+/+) were treated or not, as indicated, with 10 mM of H2O2 for 15 min. H2O2 was washed out and cells were collected after the indicated time. Total RNA were prepared and analysed by QPCR after reverse transcription. The amounts of Hsp70 cDNA were divided by the amount of GAPDH cDNA and calculated relative to 1 for untreated cells. The mean and SD from 3 independent experiments are shown.
(0.07 MB PPT)
