# Correction: The E1A-Associated p400 Protein Modulates Cell Fate Decisions by the Regulation of ROS Homeostasis

**DOI:** 10.1371/annotation/cc3111d5-d475-43ea-8d7f-f8b477b27164

**Published:** 2010-07-14

**Authors:** Lise Mattera, Céline Courilleau, Gaëlle Legube, Takeshi Ueda, Rikiro Fukunaga, Martine Chevillard-Briet, Yvan Canitrot, Fabrice Escaffit, Didier Trouche

The following text was omitted from the authors' financial disclosure: "This work was also supported by the Agence Nationale de la Recherche (ANR, http://www.agence-nationa...) [grant number ANR-06-3-13-7148]." 

