# Supplementary material for: The E1A-Associated p400 Protein Modulates Cell Fate Decisions by the Regulation of ROS Homeostasis
Source: PLoS Genet. 2010 Jun 10;6(6):e1000983. doi: 10.1371/journal.pgen.1000983 (PMC2883595; doi:10.1371/journal.pgen.1000983)
Supplement: Figure S1 — Typical experiments of siRNA-mediated silencing of p400 and/or ATM (A), p400 (B), H2A.Z (C), Hsp70 (D), and FANCA (E) in U2OS cells. U2OS cells were transfected as described throughout the manuscript. 48 hours later, total cell extracts or total RNA were prepared and analysed by western blot using specific antibodies or by QPCR after reverse transcription, respectively. The amounts of specific cDNA were divided by the amount of GAPDH cDNA and calculated relative to 1 for cells transfected by the control siRNA. A typical result is shown for each experiment and error bars stand for the variation between the three Q-PCR replicates. (2.19 MB PPT) [file pgen.1000983.s001.ppt]

## Slide 1
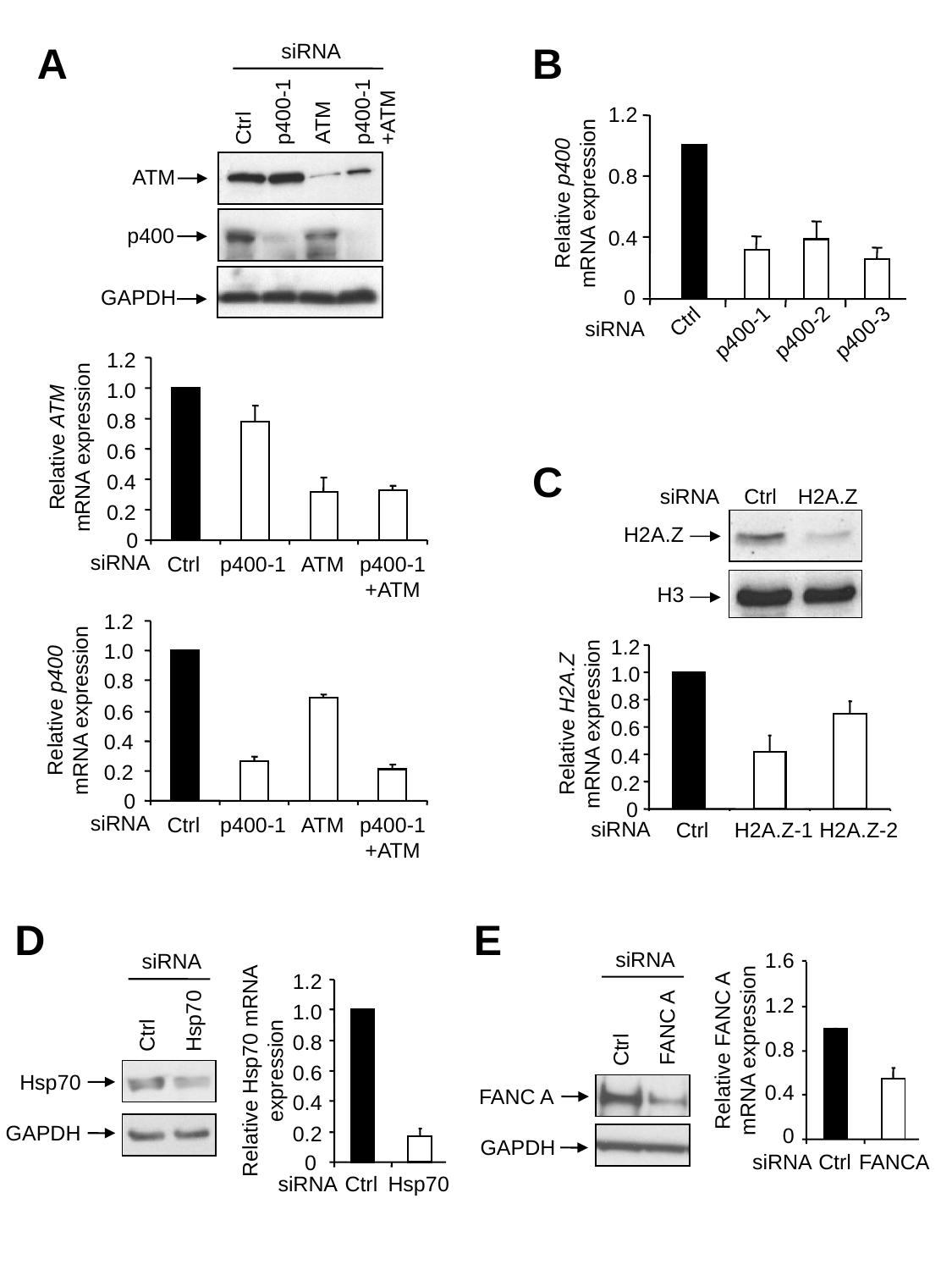

A
B
siRNA
p400-1
ATM
Ctrl
ATM
p400
GAPDH
p400-1
+ATM
1.2
0.8
Relative p400 mRNA expression
0.4
0
Ctrl
siRNA
p400-1
p400-2
p400-3
1.2
1.0
0.8
Relative ATM mRNA expression
0.6
0.4
0.2
0
p400-1
+ATM
Ctrl
p400-1
ATM
siRNA
C
siRNA
Ctrl
H2A.Z
H2A.Z
H3
1.2
1.0
0.8
Relative p400 mRNA expression
0.6
0.4
0.2
0
Ctrl
p400-1
ATM
p400-1
+ATM
siRNA
1.2
1.0
0.8
Relative H2A.Z mRNA expression
0.6
0.4
0.2
0
Ctrl
H2A.Z-1
H2A.Z-2
siRNA
D
E
siRNA
FANC A
Ctrl
FANC A
GAPDH
siRNA
Hsp70
Ctrl
Hsp70
GAPDH
1.6
1.2
Relative FANC A
mRNA expression
0.8
0.4
0
Ctrl
FANCA
siRNA
1.2
1.0
0.8
Relative Hsp70 mRNA
expression
0.6
0.4
0.2
0
siRNA
Ctrl
Hsp70
