# Supplementary material for: The E1A-Associated p400 Protein Modulates Cell Fate Decisions by the Regulation of ROS Homeostasis
Source: PLoS Genet. 2010 Jun 10;6(6):e1000983. doi: 10.1371/journal.pgen.1000983 (PMC2883595; doi:10.1371/journal.pgen.1000983)
Supplement: Figure S3 — Validation, by RT-QPCR, of DNA microarray data obtained after siRNA-mediated silencing of p400 in U2OS cells. U2OS cells were transfected as described throughout the manuscript. 48 hours later, total RNA were prepared and analysed by QPCR after reverse transcription. The amounts of specific cDNA were divided by the amount of GAPDH cDNA and calculated relative to 1 for cells transfected by the control siRNA. p400 knockdown efficiency (A) and results for two increased (B) and two decreased (C) genes are shown. Error bars stand for the variation between the three independent replicates. (0.13 MB PPT) [file pgen.1000983.s003.ppt]

## Slide 1
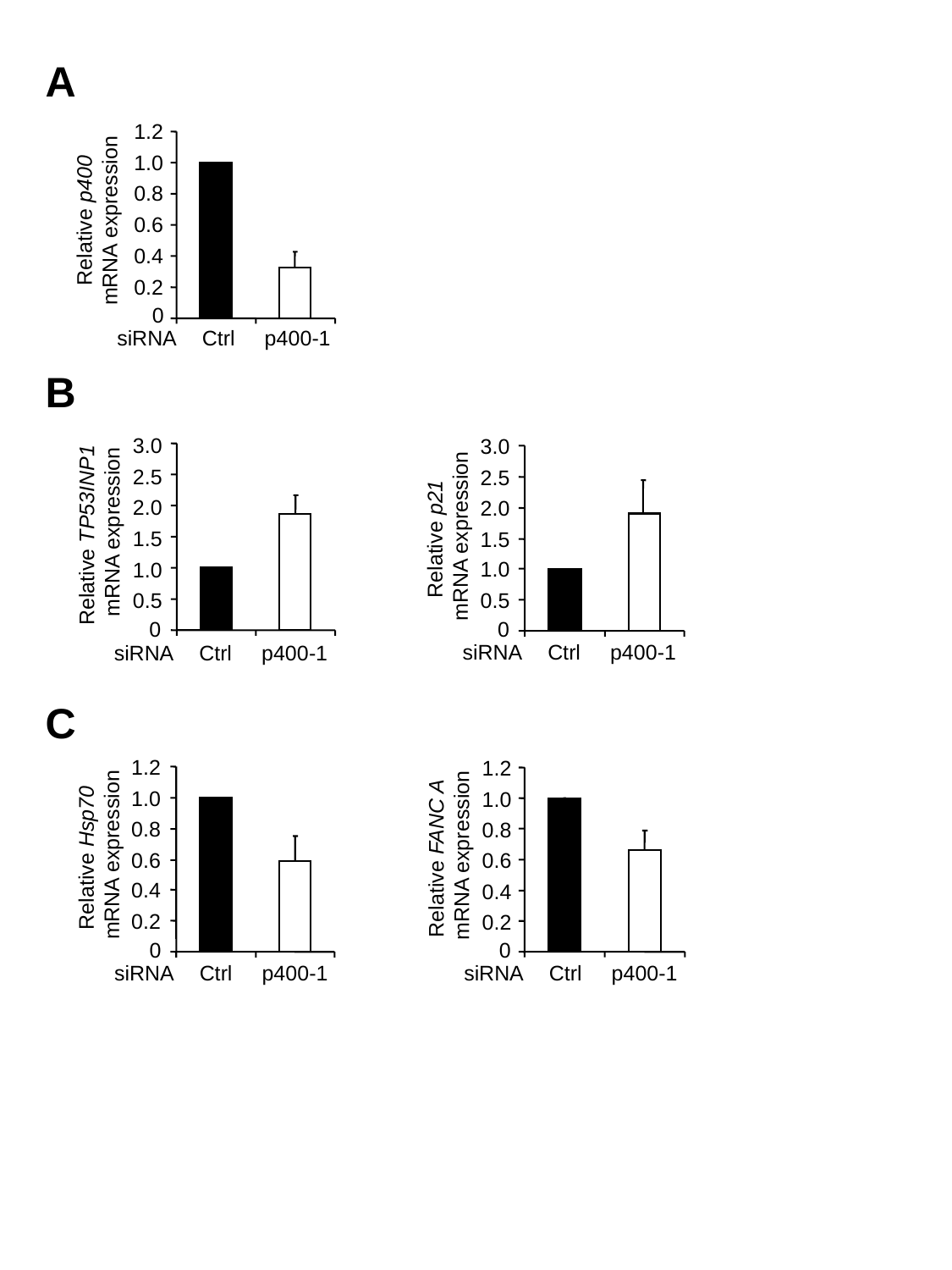

A
1.2
1.0
0.8
Relative p400 mRNA expression
0.6
0.4
0.2
0
siRNA
Ctrl
p400-1
B
3.0
2.5
2.0
Relative TP53INP1
 mRNA expression
1.5
1.0
0.5
0
siRNA
Ctrl
p400-1
3.0
2.5
2.0
Relative p21
 mRNA expression
1.5
1.0
0.5
0
siRNA
Ctrl
p400-1
C
1.2
1.0
0.8
Relative Hsp70
 mRNA expression
0.6
0.4
0.2
0
siRNA
Ctrl
p400-1
1.2
1.0
0.8
Relative FANC A
 mRNA expression
0.6
0.4
0.2
0
siRNA
Ctrl
p400-1
