# Supplementary figures and images for: The E1A-Associated p400 Protein Modulates Cell Fate Decisions by the Regulation of ROS Homeostasis
Source: PLoS Genet. 2010 Jun 10;6(6):e1000983. doi: 10.1371/journal.pgen.1000983 (PMC2883595; doi:10.1371/journal.pgen.1000983)

## Slide 1
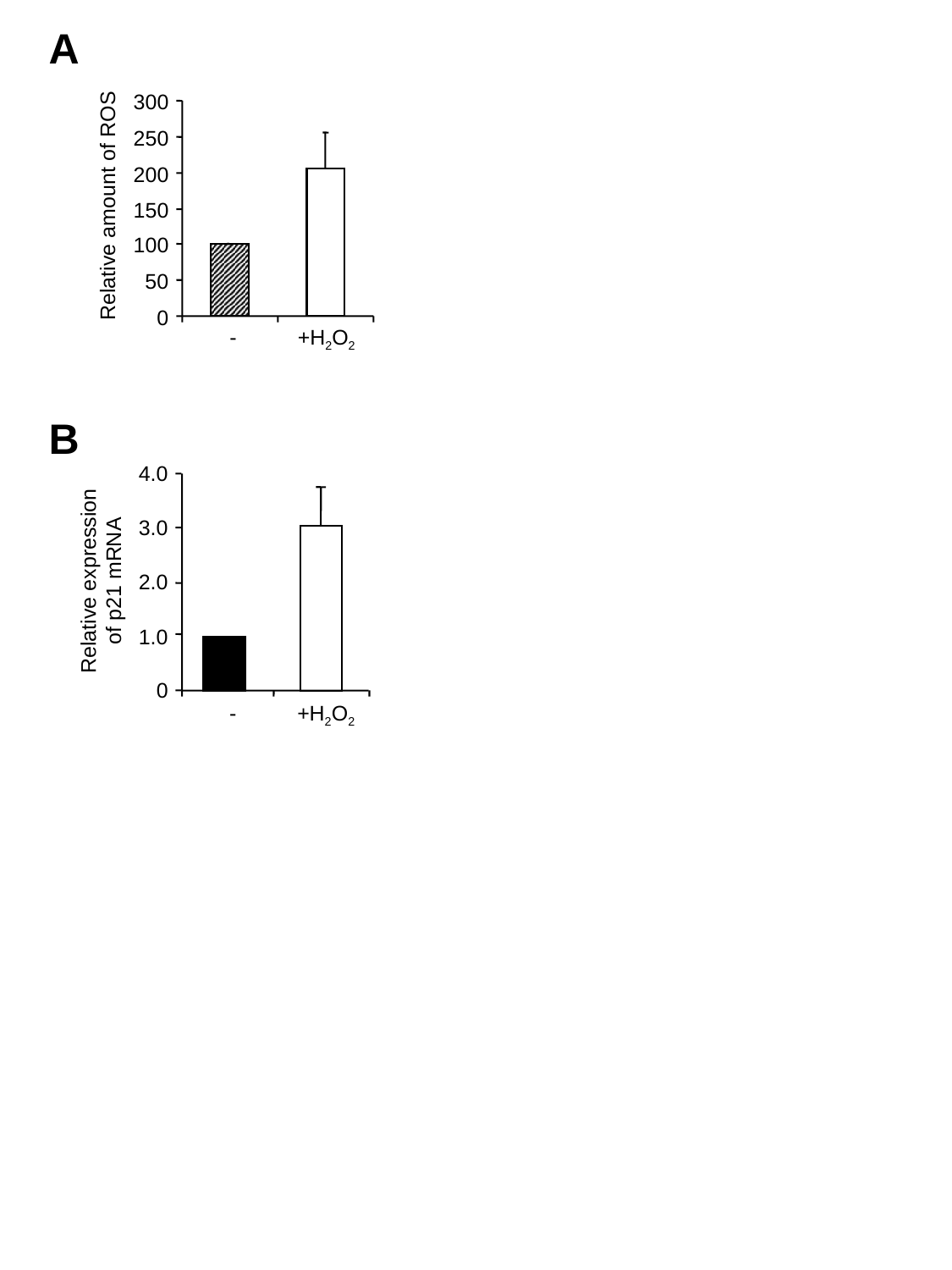

A
300
250
200
Relative amount of ROS
150
100
50
0
 -
+H2O2
B
4.0
3.0
Relative expression
of p21 mRNA
2.0
1.0
0
 -
+H2O2

Supplement: Figure S4 — Induction of ROS and p21 mRNA in U2OS by H2O2 treatment. (A) U2OS cells were treated for 15 minutes using 10 mM H2O2 in PBS. After washing with PBS, cells were incubated with the FDA probe for 15 minutes, harvested and analyzed by flow cytometry. Error bars stand for the variation between the three independent replicates. (B) U2OS cells were treated for 15 minutes using 5 mM H2O2 in PBS. After washing with PBS, cells were incubated in serum-complemented DMEM medium for 6 h at 37°C, harvested and total mRNA were extracted. After reverse-transcription, QPCR using p21 primers was performed. Error bars stand for the variation between the three independent replicates. (0.07 MB PPT) [file pgen.1000983.s004.ppt]

## Slide 1
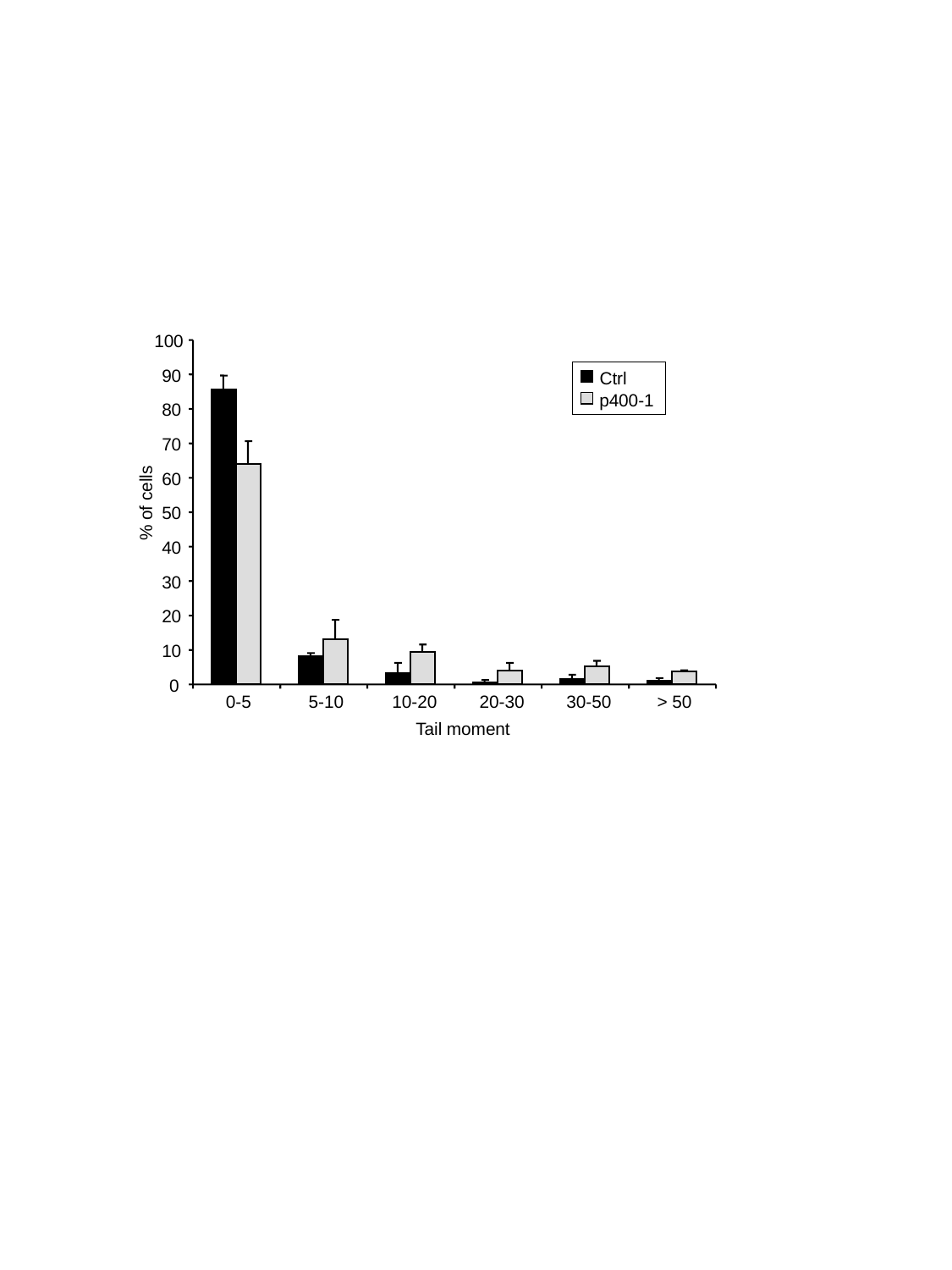

100
90
Ctrl
p400-1
80
70
60
% of cells
50
40
30
20
10
0
0-5
5-10
10-20
20-30
30-50
> 50
Tail moment

Supplement: Figure S5 — Representative experiment of Comet tails analysis. U2OS cells were transfected with siRNA. After 48 h, the presence of DNA damages was assayed by alkaline comet assay. Tail Moment was scored for 100 cells/slide. Cells with tail moments greater than 5 were considered as DNA damage positive cells. (0.07 MB PPT) [file pgen.1000983.s005.ppt]

## Slide 1
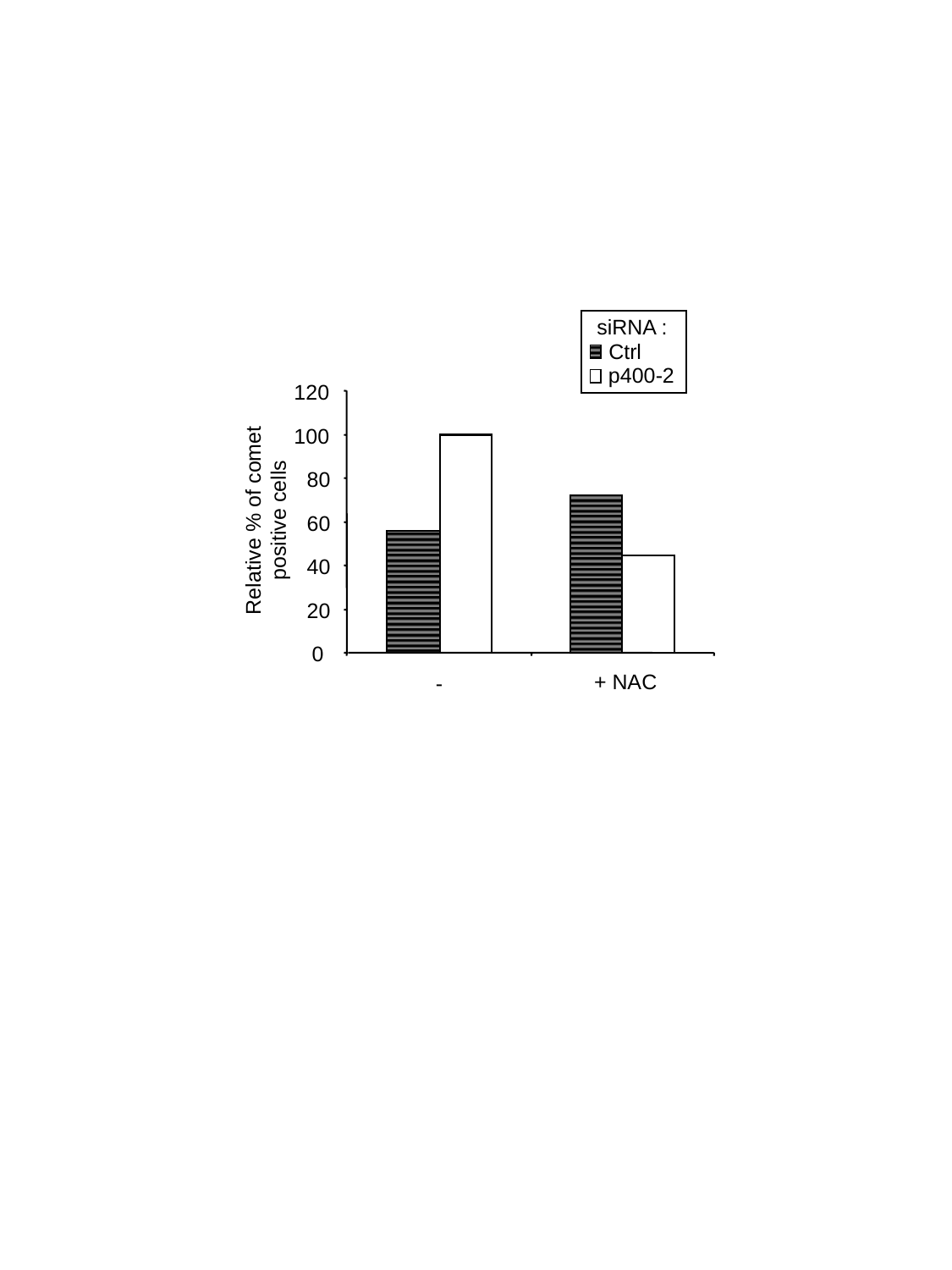

siRNA :
Ctrl
p400-2
120
100
80
Relative % of comet positive cells
60
40
20
0
 + NAC
 -

Supplement: Figure S6 — Effects of the p400-2 siRNA on DNA damage U2OS cells were transfected by the indicated siRNA as described throughout the manuscript. NAC was added, or not, 24 hours later. 48 hours following transfection, cells were harvested and subjected to a comet assay. The proportions of comet-positive cells were calculated relative to 100 for cells transfected with the p400-2 siRNA in the absence of NAC. (0.04 MB PPT) [file pgen.1000983.s006.ppt]

## Slide 1
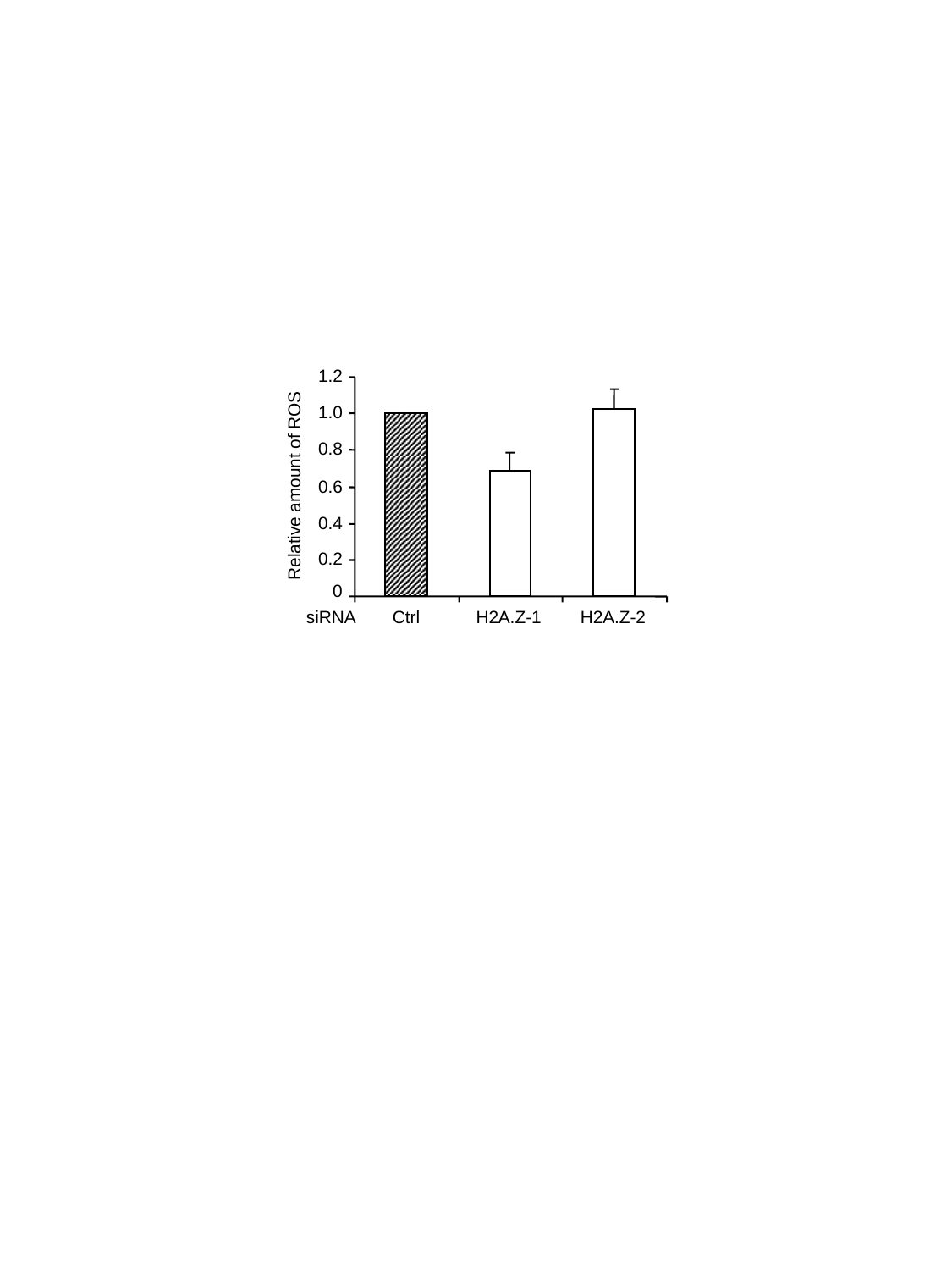

1.2
1.0
0.8
Relative amount of ROS
0.6
0.4
0.2
0
Ctrl
H2A.Z-1
H2A.Z-2
siRNA

Supplement: Figure S8 — Induction of ROS following H2A.Z knock-down. U2OS cells were transfected using two siRNAs directed against H2A.Z and ROS levels were measured 48 h later by flow cytometry. Resultswere calculated relative to 1 for cells transfected with control siRNA. The mean and SD from 3 independent experiments are shown. (0.07 MB PPT) [file pgen.1000983.s008.ppt]

## Slide 1
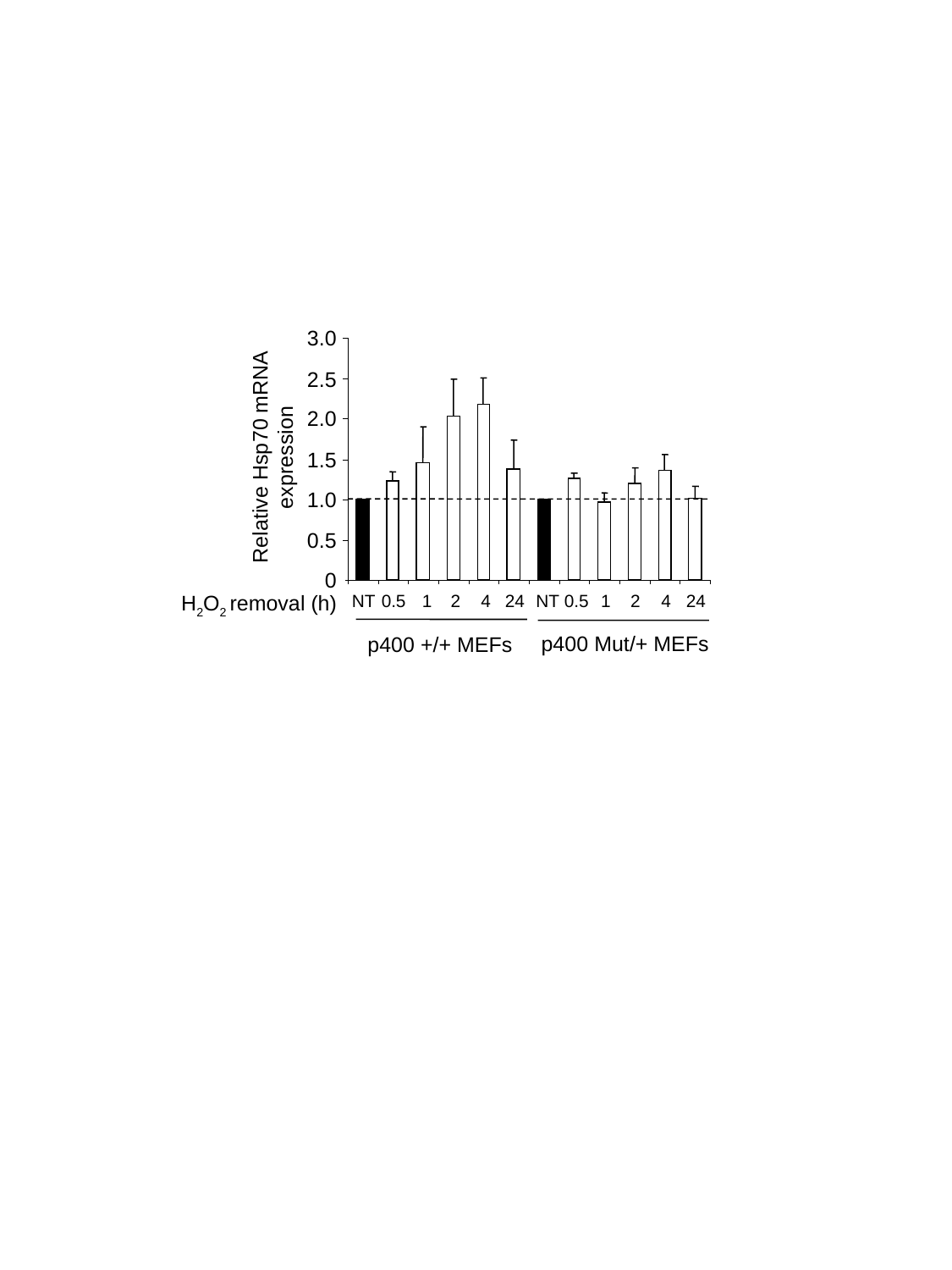

3.0
2.5
2.0
Relative Hsp70 mRNA expression
1.5
1.0
0.5
0
H2O2 removal (h)
NT
0.5
1
2
4
24
NT
0.5
1
2
4
24
p400 Mut/+ MEFs
p400 +/+ MEFs

Supplement: Figure S9 — Hsp70 mRNA expression in MEFs following H2O2 treatment. MEFs derived from heterozygous embryos in which one p400 allele was targeted (p400Mut/+)[19] or from control wild type embryos (p400+/+) were treated or not, as indicated, with 10 mM of H2O2 for 15 min. H2O2 was washed out and cells were collected after the indicated time. Total RNA were prepared and analysed by QPCR after reverse transcription. The amounts of Hsp70 cDNA were divided by the amount of GAPDH cDNA and calculated relative to 1 for untreated cells. The mean and SD from 3 independent experiments are shown. (0.07 MB PPT) [file pgen.1000983.s009.ppt]
