# Supplementary material for: The E1A-Associated p400 Protein Modulates Cell Fate Decisions by the Regulation of ROS Homeostasis
Source: PLoS Genet. 2010 Jun 10;6(6):e1000983. doi: 10.1371/journal.pgen.1000983 (PMC2883595; doi:10.1371/journal.pgen.1000983)
Supplement: Figure S7 — Characterization of silencing efficiency of p400 and ATM siRNAs in IMR90 and HCT116 cells IMR90 and HCT116 cells were transfected as described in the manuscript. siRNA-mediated silencing was checked by western blotting and reverse transcription followed by Q-PCR as described in Figure S1. A typical result is shown and error bars stand for the variation between the three Q-PCR replicates. Note that we were unable to detect full length p400 in IMR90 cells because of a co-migrating non-specific band. (2.37 MB PPT) [file pgen.1000983.s007.ppt]

## Slide 1
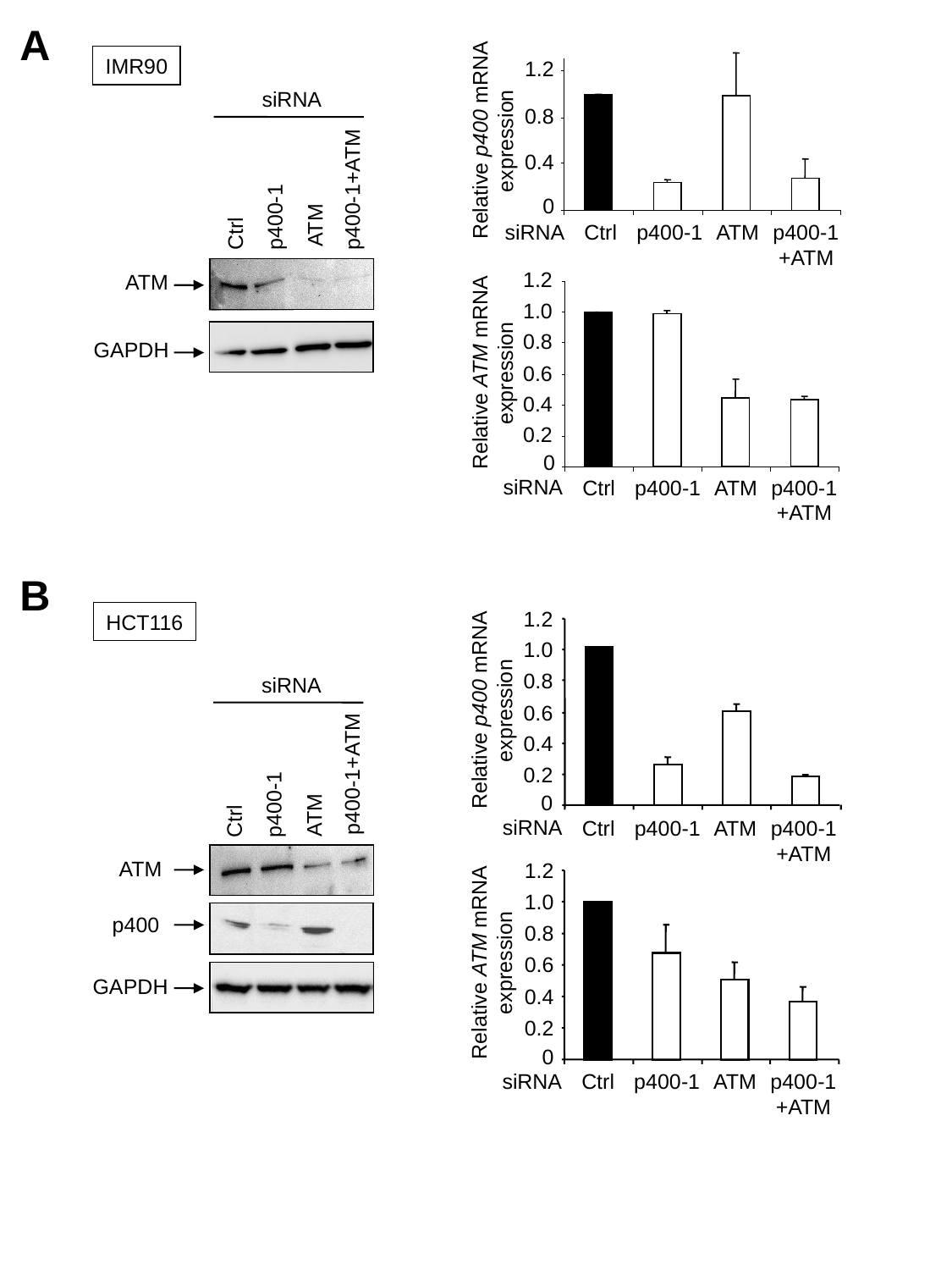

A
1.2
0.8
Relative p400 mRNA expression
0.4
0
Ctrl
p400-1
ATM
p400-1
+ATM
siRNA
IMR90
siRNA
p400-1+ATM
p400-1
ATM
Ctrl
ATM
GAPDH
1.2
1.0
0.8
Relative ATM mRNA expression
0.6
0.4
0.2
0
Ctrl
p400-1
ATM
p400-1
+ATM
siRNA
B
HCT116
siRNA
p400-1+ATM
p400-1
ATM
Ctrl
ATM
p400
GAPDH
1.2
1.0
0.8
Relative p400 mRNA expression
0.6
0.4
0.2
0
Ctrl
p400-1
ATM
p400-1
+ATM
siRNA
1.2
1.0
0.8
Relative ATM mRNA expression
0.6
0.4
0.2
0
Ctrl
p400-1
ATM
p400-1
+ATM
siRNA
